# Telomere-to-Telomere Genome Assembly of Tibetan Medicinal Mushroom *Ganoderma leucocontextum* and the First *Copia* Centromeric Retrotransposon in Macro-Fungi Genome

**DOI:** 10.3390/jof10010015

**Published:** 2023-12-27

**Authors:** Miao Wang, Guoliang Meng, Ying Yang, Xiaofang Wang, Rong Xie, Caihong Dong

**Affiliations:** 1 State Key Laboratory of Mycology, Institute of Microbiology, Chinese Academy of Sciences, Beijing 100101, China; mwang2136@gmail.com (M.W.); 18706387822@163.com (G.M.); yangy@im.ac.cn (Y.Y.); wang227529@163.com (X.W.); 2 University of Chinese Academy of Sciences, Beijing 100049, China; 3 Institute of Vegetable Sciences, Tibet Academy of Agricultural and Animal Husbandry Sciences, Lhasa 850000, China; xr-20082004@163.com

**Keywords:** *Ganoderma*, centromere, *Copia*, telomere-to-telomere

## Abstract

A complete telomere-to-telomere (T2T) genome has been a longstanding goal in the field of genomic research. By integrating high-coverage and precise long-read sequencing data using multiple assembly strategies, we present here the first T2T gap-free genome assembly of *Ganoderma leucocontextum* strain GL72, a Tibetan medicinal mushroom. The T2T genome, with a size of 46.69 Mb, consists 13 complete nuclear chromosomes and typical telomeric repeats (CCCTAA)n were detected at both ends of 13 chromosomes. The high mapping rate, uniform genome coverage, a complete BUSCOs of 99.7%, and base accuracy exceeding 99.999% indicate that this assembly represents the highest level of completeness and quality. Regions characterized by distinct structural attributes, including highest Hi-C interaction intensity, high repeat content, decreased gene density, low GC content, and minimal or no transcription levels across all chromosomes may represent potential centromeres. Sequence analysis revealed the first *Copia* centromeric retrotransposon in macro-fungi genome. Phylogenomic analysis identified that *G. leucocontextum* and *G. tsugae* diverged from the other *Ganoderma* species approximately 9.8–17.9 MYA. The prediction of secondary metabolic clusters confirmed the capability of this fungus to produce a substantial quantity of metabolites. This T2T gap-free genome will contribute to the genomic ‘dark matter’ elucidation and server as a great reference for genetics, genomics, and evolutionary studies of *G. leucocontextum*.

## 1. Introduction

*Ganoderma leucocontextum* T.H. Li, W.Q. Deng, Sheng H. Wu, Dong M. Wang and H.P. Hu, also known as ‘Zanglingzhi’ or ‘White Lingzhi’ in China, is a medicinal species that was firstly identified in 2015 [1]. It belongs to the class Agaricomycetes, order Polyporales, and family Polyporaceae. This species is primarily found in high-altitude regions of southwestern China, particularly in the Tibet Autonomous Region and Sichuan Province.

The distinct habitat contributes to the production of unique compounds. It has been reported that *G. leucocontextum* can produce an abundance of bioactive compounds such as triterpenes and ganoderols. These compounds contribute to the effectiveness of this species in treating various diseases. Twenty-four new lanostane-type triterpenoids, leucocontextins A–X, were isolated from the fruiting bodies of *G. leucocontextum* [2,3]. Sixteen new lanostane triterpenes, ganoleucoins A–P, together with 10 known tripterpenes, were isolated from the cultivated fruiting bodies of *G. leucocontextum* and some showed much strong inhibitory activity against HMG-CoA reductase [4]. Ganoderiol F purified from *G. leucocontextum* retards cell cycle progression and may serve as a potential CDK4/CDK6 inhibitor for breast cancer therapy [5]. A novel natural triterpene GL22, isolated from *G. leucocontextum*, can suppress tumor growth by altering lipid metabolism and triggering cell death [6]. However, the biosynthetic pathway and regulation of these bioactive compounds in *G. leucocontextum* are hindered by the lack of a high-quality genome sequence.

Efforts were undertaken more than two years ago to sequence the *G. leucocontextum* genome, resulting in the publication of three reference genomes. These include two draft genomes at the contig level [7,8] and one genome at the chromosome level [9]. However, this chromosome-level genome remained incomplete, with 2800 gaps. A gap-free genome is the ultimate goal of genome assembly, which brings new opportunities for the identification of unique genes and structural variations in the “dark matter” regions, such as centromeres, transposable elements (TEs), and segmental duplications [10]. More than two decades after the draft human genome, the first complete, gap-free sequence of a human genome has been published to pave the way for new insights into health and what makes our species unique [11]. Gap-free genomes have been completed in plant, such as rice [12,13], Arabidopsis [14] and watermelon [10], and fungi, such as *Ustilaginoidea virens* [15]. However, gap-free genome of mushroom has been reported seldomly.

In this study, we assembled a T2T gap-free genome of *G. leucocontextum* using monokaryon of a commercial cultivar by long-read sequencing technology on Pacific Biosciences (PacBio, Menlo Park, CA, USA) sequencing platform, Hi-C scaffolding techniques, and manual polishing. The T2T gap-free reference genome was assessed by BUSCO, genome coverage, telomere at both ends and mapped k-mers analyses. Based on the gap-free genome, the positions of centromeres were predicted and the centromere sequence analysis revealed the first *Copia* centromeric retrotransposon in macro-fungi genome. Phylogenomic and evolutionary analysis revealed the phylogenetic relationship and species divergence times. The secondary metabolic clusters were predicted with plenty of terpene gene clusters. This represents the first report of a gap-free T2T genome, specifically within the *Polyporaceae* family.

## 2. Material and Methods

### 2.1. Fungal Strains and DNA Preparation

**Figure 1:**
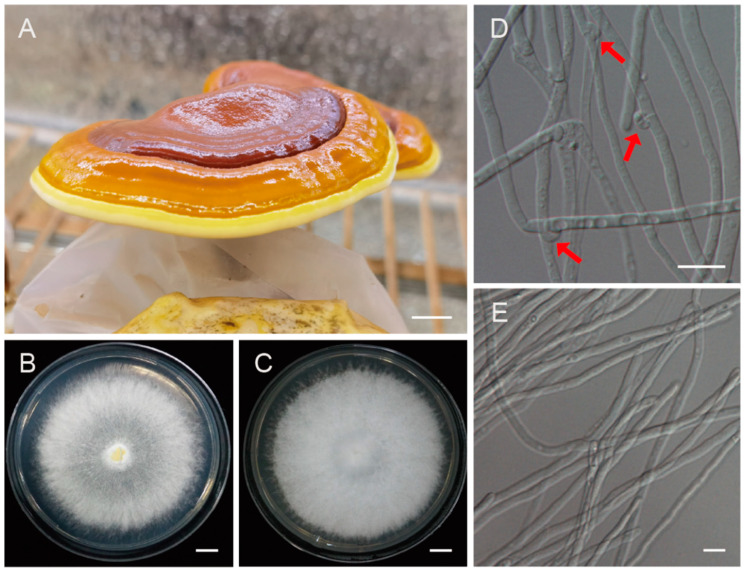
The fruiting bodies of *Ganoderma leucocontextum* and the monokaryotic strain used for genome sequencing. (**A**) The cultivated fruiting body of *G. leucocontextum*. (**B**,**D**) Colony and heterokaryotic mycelia with clamp connections. Red arrows indicated the clamp connections. (**C**,**E**) Colony and vegetative mycelia of the monokaryotic strain GL72. Bars: (**A**–**C**) = 1 cm; (**D**,**E**) = 10 μm.

The dikaryon strain *G. leucocontextum* 1396 (CGMCC 5.2224) is a culture of commercial cultivar in Tibet Autonomous Region and was provided by associate Prof. Rong Xie from the Institute of Vegetable Sciences, Tibet Academy of Agricultural and Animal Husbandry Sciences. Monokaryons were isolated by the protoplast monokaryogenesis technique [9]. The monokaryotic strains without any clamp connection were selected under 600× magnification using an optical microscope (Eclipse 80i, Nikon, Tokyo, Japan) and subcultured individually on PDA plates at 25 °C (Figure 1). The monokaryotic strain GL72 was cultured on PDA plates for 7 d and the vegetative mycelia were collected. High-quality genomic DNA was extracted using the QIAGEN^®^ Genomic kit (Qiagen, Dusseldorf, Germany) following the manufacturer’s protocol.

### 2.2. Library Construction and Sequencing

Multiple sequencing techniques including Next Generation Sequencing (NGS), PacBio and Hi-C were applied to develop a reference genome assembly for the monokaryotic strain GL72. First, an NGS library with an average insertion size of 350 base pairs (bp) was created using the MGISEQ standard protocol. Additionally, a Hi-C library was generated using the *Dpn*II enzyme according to a previously published protocol [16]. Briefly, the freshly collected mycelia were fixed by crosslinking with 2% formaldehyde, and then ground and resuspended in a buffer solution. Digestion was carried out using 100 units of *Dpn*II, and incubation with biotin-14-dCTP was employed for labeling. The ligated DNA was sheared into 300–600 bp fragments and then was blunt-end repaired and A-tailed, followed by purification through biotin-streptavidin-mediated pull-down. Finally, both the NGS and Hi-C libraries were quantified and sequenced on the MGISEQ2000 platform. A 20-kb library was also constructed following PacBio’s standard method, and its quality was evaluated using the Agilent 2100 Bioanalyzer (Agilent Technologies, Santa Clara, CA, USA). Single-molecule real-time (SMRT) cells were sequenced on the PacBio Sequel II sequencing platform. All these sequencing services were provided by Nextomics Biosciences Co., Ltd. (Wuhan, China).

For the transcriptome analysis, *G. leucocontextum* strain 1396 was cultured on PDA media and the mycelia were collected for RNA extraction. Three libraries were generated using the NEB Next Ultra RNA Library Prep Kit for Illumina (NEB, Ipswich, MA, USA) and sequenced on an Illumina HiSeq X-ten platform (Illumina Inc., San Diego, CA, USA) by Nextomics Biosciences Co., Ltd. (Wuhan, China).

### 2.3. Genome Heterozygosity Estimation

To eliminate low-quality reads, adapter sequences, and reads containing poly-N, the raw paired-end reads obtained from MGI sequencing were subjected to preprocessing using the fastp v0.20.0 [17] with default parameters. The following filtering parameters were applied: --n_base_limit 10 --adapter_trim_min 10 --adapter_trim_max 10 --qualified_quality_phred 5.

To gain insights into the genomic characteristics, Illumina DNA data were analyzed using k-mer analysis before genome assembly. The quality-filtered reads were subjected to 17-mer frequency distribution analysis using KMC [18]. The 17-mer depth distribution was analyzed using GCE [19] and FindGSE_v1.94.R software [20] to estimate the genome size and the heterozygosity.

### 2.4. Genome De Novo Assembly, Polishing and Quality Assessment

For the PacBio assembly, low-quality regions and adapter sequences in raw reads were removed using Smrtlink v7.0, and HiFi reads were then generated using CCS software (https://github.com/pacificbiosciences/unanimity, accessed on 22 November 2022) with the default parameter. Reads length less than 10 Kb were filtered using SAMtools v1.17 [21]. These filtered, long and highly accurate HiFi reads were assembled using Hifiasm v0.13 [22] with default parameters to generate a draft contig genome. 

For the Hi-C assembly, Hi-C data were used to anchor hybrid contigs onto the chromosomes. Sequences shorter than 30 bp and adapter sequences in the Hi-C library were filtered out using fastp v0.20.0 [17]. The clean paired-end reads were then mapped to the draft assembled sequence using Bowtie2 v2.3.2 [23] to obtain unique mapping paired-end reads. HiC-Pro v2.8.1 [24] was used to identify valid interaction-paired reads and filter out invalid read pairs. The contigs were further clustered, ordered, and oriented onto chromosomes by LACHESIS [25]. Any placement and orientation errors in the discrete chromatin interaction patterns were then manually corrected. Finally, a heatmap of genomic interactions was plotted. 

**Figure 2:**
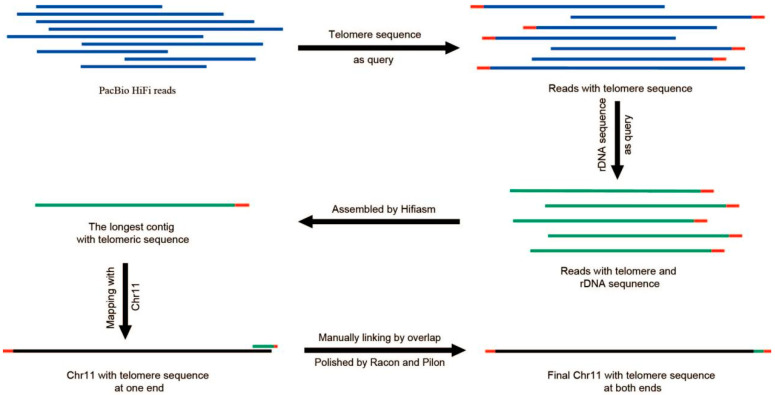
Schematic showing the assembly of the telomere of Chr 11. The blue represented PacBio reads, the red represented telomere repeats, the green represented rDNA sequence, and the black represented genome sequence.

To patch the missing telomeres, a manual identification process was conducted on telomeric reads (Figure 2). Specifically, six-base telomere repeats (‘CCCTAA’ and ‘TTAGGG’) were employed as queries to extract pertinent sequences from PacBio HiFi reads. The extracted sequences were reverse-complemented and consolidated into a comprehensive library. The specific sequences associated with the absent telomeres were detected from the library. Subsequently, these identified sequences were employed for assembly using Hifiasm with default parameters, and based on the assembly results, the telomere-missing chromosomes were completed. Both Racon [26] and Pilon [27] were utilized in three iterative rounds of polishing. This iterative process involved refining the consensus sequences and resolving any potential errors, resulting in an improved assembly quality.

All the PacBio HiFi reads were aligned to the T2T assembly using minimap2 v2.1 [28] to identify local coverage-anomalous regions on the basal GL72 assembly. Sambamba 0.6.6 [29] was used to calculate the average depths for all 10 kb bins of the genome. The bins with depth lower than 80 (genome-wide average: 248) were identified as local coverage anomalies. We used Integrative Genomics Viewer (IGV) [30] for visualizing and analyzing data to investigate the causes of local coverage anomalies. Based on the results of multiple software assemblies [Canu v2.2 [31], Hifiasm [22], Wtdbg2 v2.5 [32]], manual adjustments were made to genomic regions displaying coverage anomalies by examining the original reads.

The integrity of the chromosome-level genome assembly was estimated by BUSCO v4.0.3 [33] using basidiomycota_odb10 lineage data (https://busco-data.ezlab.org/v5/data/lineages/basidiomycota_odb10.2020-09-10.tar.gz, accessed on 23 February 2023). The accuracy of the final assembly was estimated from mapped k-mers via Merqury v1.3 [34]. In brief, the optimal k-mer size was determined as 17 based on the final assembly size. NGS data were utilized to generate a k-mer database with the selected k-mer size. In the Merqury anlysis, each k-mer in the GL72 assembly was assessed for its presence in the total k-mer database generated from NGS data.

### 2.5. Prediction of Centromere Position

The positions of centromeres were predicted by combining the Hi-C heatmap and sequence characteristic analyses using TBtools [35]. High-frequency interchromosomal contacts occurred within the pericentromeric and subtelomeric regions, including centromeres and telomeres [36]. 

To further confirm the accuracy of the predicted centromeres, an algorithm based on OE intensity (observed/expected interaction frequency) of Hi-C data was used to predict the centromere position of each chromosome following the method used in the giant panda (*Ailuropoda melanoleuca*) [37] with some modifications. The Hi-C dataset was processed using Juicer v2.0 [38], and normalized contact matrices at 10 K resolution were constructed by filtering abnormal, duplicate, and low-quality (Mapping quality < 30) alignments. We then extracted the symmetric matrix with a step of 10 Kb and calculated the OE intensity. Based on the OE intensity, the centromere region of each chromosome was predicted. 

The differences between long and short arms and arm ratios of each chromosome were calculated following the classic method [39].

### 2.6. Copy number Estimation of rDNAs

To identify the Ribosomal DNA (rDNA) regions within the genome, 28S rRNA (GeneBank ID: NG_042623.1), 5.8S rRNA (GeneBank ID: NR_111007.1), and 18S rRNA (GeneBank ID: NG_063315.1) from *Saccharomyces cerevisiae* were utilized as a BLAST query. The copy number of rDNA in the genome was estimated using the blast-based method [40] with PacBio HiFi data. In detail, the rDNA sequences present in the data were identified by blastn v2.9.0 [41] using the following parameters: -task megablast-max_hsps 5000-max_target_seqs 100,000. The copy number of rDNA was then calculated using the following formula:

$$copy\;number=\frac{total\;length\;of\;rDNA}{\left(average\;genome\;coverage\right)\times (length\;of\;rDNA\;repeat\;unit)}$$

### 2.7. Repeat Annotation, Gene Prediction and Gene Function

Tandem repeats in GL72 genome were predicted by GMATA v2.2 [42] and TRF v 4.07b [43]. To identify transposable elements (TEs), MITE-hunter [44] was utilized to detect MITEs (Miniature Inverted-repeat Transposable Elements) in the genome, resulting in the creation of a MITE library. LTR (Long Terminal Repeat) elements were detected by LTR_finder [45] and LTR_harvest [46], followed by construction of an LTR library using LTR_retriever [47]. We integrated the two libraries to create a TE library file (TE.lib). To form a comprehensive library, we merged TE.lib with the de novo library (RepMod.lib) generated by RepeatModeler [48]. Repbase [49] was also included in the library. Using this final library, RepeatMasker [50] was employed to search for repetitive sequences across the entire genome. The presence of unknown repetitive sequences in the predicted results was then classified using DeepTE [51].

Gene prediction was performed by combining ab initio, homology-based, and transcriptome-based approaches. Augustus V3.3.1 [52] was used for ab initio prediction. For the homology-based prediction, protein sequences from five fungi, *G. sinense* ZZ0214-1 [53], *Dichomitus squalens* LYAD-421 SS1 [54], *Lentinus tigrinus* ALCF2SS1-6 and ALCF2SS1-7 [55] and *Polyporus brumalis* [56] were used to construct gene models by GeMoMa v.1.6.2 [57]. For the transcript evidence approach, RNA-seq data were mapped to the genome assembly using PASA v2.3.3 [58] to define a more accurate gene structure. EVM [59] was utilized to integrate these collected data, resulting in a non-redundant gene set.

To functionally annotate the predicted genes, Blastp v2.7.1 [41] was used to align the protein-coding sequence to the public databases, such as Non-Redundant Protein Database (NR), Kyoto Encyclopedia of Genes and Genomes (KEGG), Eukaryotic Orthologous Groups of Proteins (KOG), and Swissprot databases. InterproScan 5.32-71.0 [60] was used to obtain the annotations of biological processes (BP), cellular components (CC), and molecular functions (MF) based on gene ontology (GO) using default parameters.

### 2.8. Phylogenomic and Evolutionary Analyses

Phylogenomic analyses were conducted using 15 fungal genomes (Table S1), including two fossil record fungal species (*Coprinopsis cinerea* and *Laccaria bicolor*) [54]. Single-copy orthologous genes were identified using OrthoFinder version 2.5.4 [61]. Protein sequences of single-copy genes were aligned using Muscle version 3.8.31 [62] with default parameters. The conserved sequences were selected using Gblocks version 0.91b [63] and concatenated into supermatrix using seqkit version 2.2.0 [64]. A maximum likelihood-based phylogenetic tree was built using RAxML-NG version 0.9.0 [65], with the amino acid replacement matrix LG + I + G + F, as selected by ProtTest version 3.4.2 [66]. One thousand bootstrap replicates were used, and the best tree was shown in FigTree v1.4.4 (https://github.com/rambaut/figtree/, accessed on 20 May 2023). The species divergence times were estimated by Beast version 2.67 [67] with an approximate likelihood method based on fossil calibrations for *L. bicolor* and *C. cinerea* (59.3–108.4 MYA).

The phylogenetic relationships and gene families obtained above were used to calculate the expansion or contraction of gene families using CAFE v.4.2.1 [68]. A Monte Carlo resampling procedure was applied to a random sample of 1000, and a *p*-value cutoff of 0.05 was used. 

Unique gene families among GL72, *G. sinense*, *G. sichuanense*, and *G. tsugae* were identified using OrthoFinder version 2.5.4 [61]. The KEGG enrichment of expanded, contracted and unique gene families were then analyzed using TBtools.

### 2.9. Whole-Genome Collinearity Analysis

In order to investigate the genomic structure variations and conservation of GL72, we employed two cultivated *Ganoderma* species widely in China, *G. sichuanense* and *G. tsugae*, whose genomes were published in a recent study with high quality [9] for whole-genome collinearity analysis by Mummer version 4.0.0beta2 [69]. Low quality collinear fragments with alignment coverage and identity percentage less than 80 were removed, and the filtered data was plotted using package *RIdeogram* [70] in R.

### 2.10. Prediction of the Secondary Metabolite Gene Clusters

Secondary metabolite gene clusters were predicted with fungal AntiSMASH 3.0 (https://fungismash.secondarymetabolites.org/, accessed on 11 February 2023). 

## 3. Results

### 3.1. A Telomere-to-Telomere (T2T) Gap-Free Assembly of G. leucocontextum Strain GL72

To generate high-quality genome assembly, the protoplast-derived monokaryotic strain GL72 was used for genome sequencing (Figure 1) by both the MGISEQ2000 and PacBio SEQUEL II platforms. A total of 4.84 Gb (~48×) MGI pair-end reads and 12.08 Gb (~248×) PacBio HiFi reads were generated. The predicted genome size ranges from 46.48 to 59.34 Mb. There was no apparent heterozygous peak, and the heterozygosity was low at 0.094% based on the k-mer analysis (Figure S1). 

**Figure 3:**
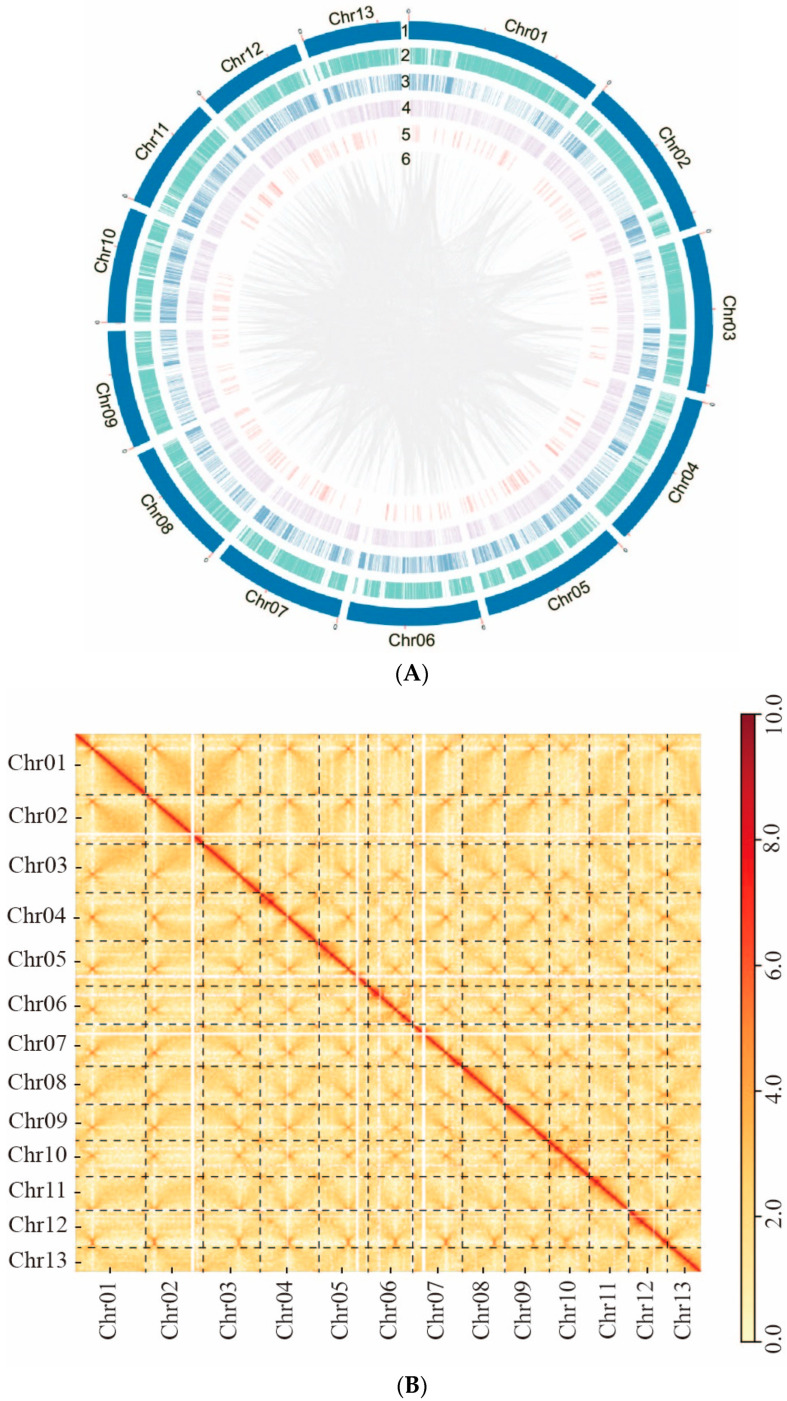

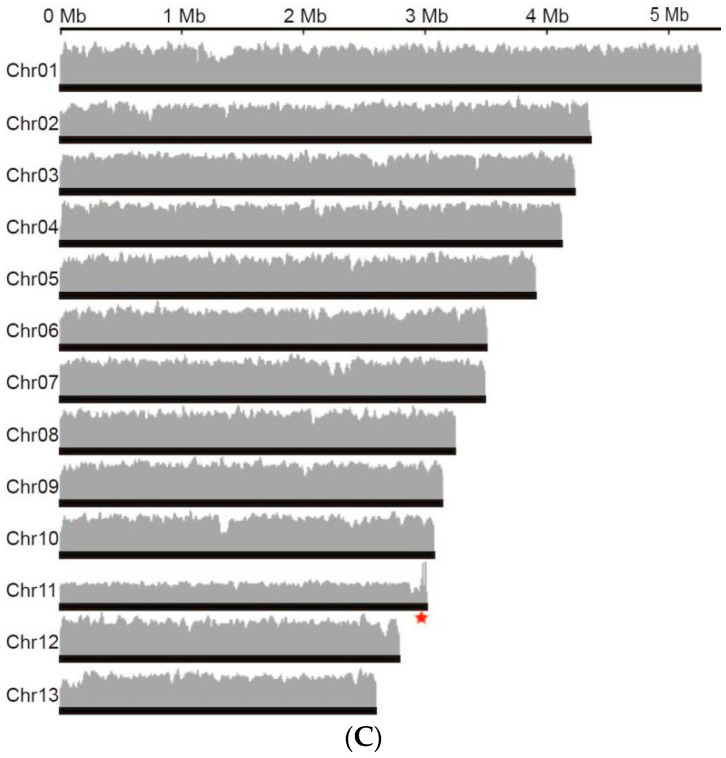
The genome assembly of *Ganoderma leucocontextum* GL72. (**A**) Circos graph of the characteristics of *Ganoderma leucocontextum* genome. (1) 13 chromosomes. (2) Gene density. (3) Transposable elements. (4) Tandem Repeats. (5) Non-coding RNA. (6) Large fragment duplication (>1 kb). (**B**) Hi-C interaction heatmap of all 20 kb bins. Highest Hi-C interaction intensity in each chromosome represented potential centromere position. (**C**) Genome coverage of GL72, the red star indicated the rDNA region.

**Figure 4:**
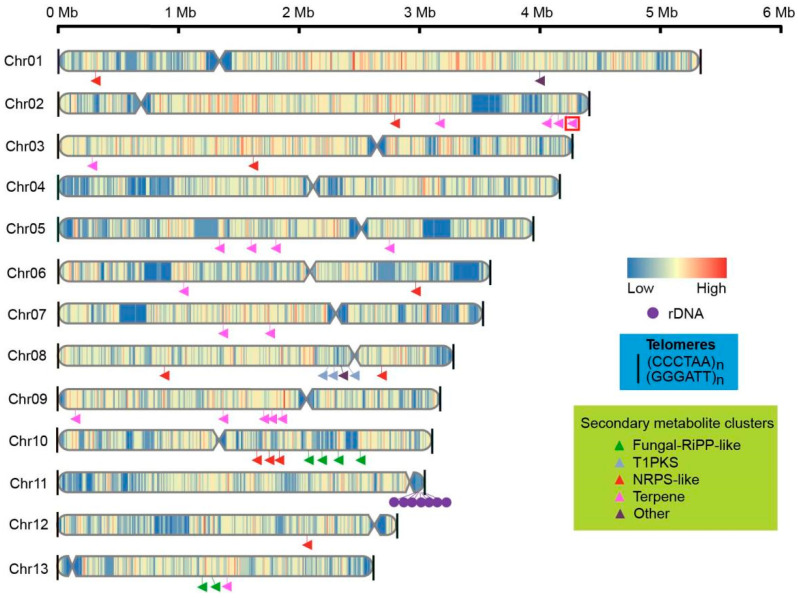
Chromosome structures, including the telomeres (black line), centromeres (indentations), and distribution of genes and secondary metabolite clusters on chromosomes of GL72. The triangular arrowheads indicated the secondary metabolite clusters while the unique clusters of *G. leucocontextum* were highlighted with a red frame.

As a result of the contig-level genome assembly, an assembled size of 46.22 Mb and a total of 16 contigs were obtained. Then, with 7.38 Gb of clean Hi-C data (~160×), sequences were located on 13 chromosomes by LACHESIS agglomerative hierarchical clustering and a reference genome was thus generated. These 13 chromosomes were denoted with Arabic numerals (i.e., Chr 01 to Chr 13), from largest to smallest (Figure 3A and Figure 4). In addition to the nuclear genome, we assembled a circular mitochondrial genome with size of 89,684 bp and GC content of 27.05%. It comprises fifteen protein-coding genes, 2 ribosomal RNA genes, and 25 tRNA genes (Figure S2).

Then, genome coverage analysis was performed and two distinct regions with localized coverage anomalies were observed in Chr 06 and Chr 07 (Figure S3). In the case of Chr 06, when we used TBtools to extract the corresponding sequences, we found a repetitive unit of approximately 10 K with an initial estimation of 8 copies. However, after conducting coverage analysis, it was determined that the accurate copy number for this region is 5. This anomaly could be attributed to an erroneous estimation by the software. The adjustment was further validated by the assembly obtained using the Hifiasm software, which incorporated HiFi and Hi-C data. Additionally, subsequent genome coverage analysis provided further evidence supporting the accuracy of the corrected copy number (Figure 3C). For Chr 07, inspection using IGV revealed a predominance of reads with a mapping quality of 0, indicating possible assembly errors. After employing the corrected assembly results obtained from Canu, comprehensive analysis of genome coverage consistently confirmed its accuracy (Figure 3C).

A heatmap generated from Hi-C data indicated that all bins could be categorized into 13 chromosomes (Figure 3B). In each group, the interaction intensity at the diagonal exceeded that of off-diagonal position, signifying strong interactions between adjacent sequences. No noticeable noise was observed outside the diagonal, indicating a satisfied genome assembly. 

Using the rDNA sequence of *Saccharomyces cerevisiae* as a query, we identified the sequence of a single rDNA unit, which was determined to be 5701 bp in length. Through a blast analysis, we estimated the total length of the matching region in PacBio reads with rDNA to be 102,247,170 bp and the copy number of rDNA was calculated to be 72.

Telomeric repeats (CCCTAA)n were identified at both ends of 12 chromosomes, while only Chr 11 possessed a telomeric sequence at one end. The terminal region of Chr 11 sequence was identified as rDNA repetitive sequences by blast analysis, suggesting that the incomplete assembly is primarily attributed to these repetitive sequences. To address the issue of the missing telomere, a manual identification process was conducted on the telomeric reads (Figure 2). Finally, telomeric repeats were detected at both ends of all 13 chromosomes (Figure 4).

The final assembled genome had a size of 46.69 Mb with 13 chromosomes, exhibiting good continuity with an N50 value of 3.54 Mb (Table 1). The longest chromosome was 5.26 Mb, while the shortest was 2.59 Mb in length (Figure 4, Table S2).

### 3.2. Evaluation of the Final T2T GL72 Assembly

The quality of the assembled genome was evaluated using BUSCO v 4.1.3. A total of 1758 genes, approximately 99.7% of the complete BUSCOs, can be found in the basidiomycota_odb10 database with conserved single-copy homologous genes, indicating a high level of quality and completeness (Table S3). 

A total of 604,780 filtered HiFi reads were remapped to the T2T assembly using the minimap2 [28]. 574,965 reads (95.07%) were aligned with the T2T reference genome and 29,810 (4.92%) were successfully mapped to the mitochondrial genome. The remaining unmapped 5 reads (<0.01%) may be due to bacterial contaminants or untraceable origins. 

A uniform coverage across nearly all genomic regions revealed by Sambamba 0.6.6 [29] confirmed the overall accuracy of the assembly (Figure 3C). The accuracy of the final assembly was also estimated from mapped k-mers via Merqury v1.3 [34]. Out of 34,238,721 k-mers, only 4471 k-mers were exclusively detected in the assembly. The quality value (QV) score was calculated using the formula: −10 × log (1 − (1 − 4471/34,238,721)^(1/17)^) = 54.1454. The overall base accuracy of the T2T GL72 assembly was estimated to be 99.9992% (QV score, 54.1454) based on mapped k-mers using the formula: 100 − (10^(54.1454/−10)^) × 100 = 99.9992%. 

Our newly sequenced *G. leucocontextum* genome represents the first T2T gap-free genome assembly of *G. leucocontextum.* When compared to three previously published genomes (Table 1), our assembly showed significant improvements. The assembly of *G. leucocontextum* strains Dai 12418 [8] and HMGIMI160015 [7] consisted of 843 contigs with N50 value of 0.21 Mb and 58 contigs with N50 of 3.1 Mb, respectively. Notably, the N90 value of strain HMGIMI160015 was only 0.48 Mb, much lower than 2.8 Mb observed in strain GL72. In comparison to the chromosome-level assembly of strain CCMJ4170 [9], which contained a total of 2800 “N” sites and only 14 telomeres in its genome (Table S4), our assembly exhibited a higher level of continuity and integrity. The N50 value, 3.39 Mb, was also lower than that of strain GL72. Overall, our newly sequenced *G. leucocontextum* genome surpasses the previously published genomes in terms of contiguity and assembly quality. 

### 3.3. Prediction of Centromere Regions of Chromosome and Sequence Analysis 

**Figure 5:**
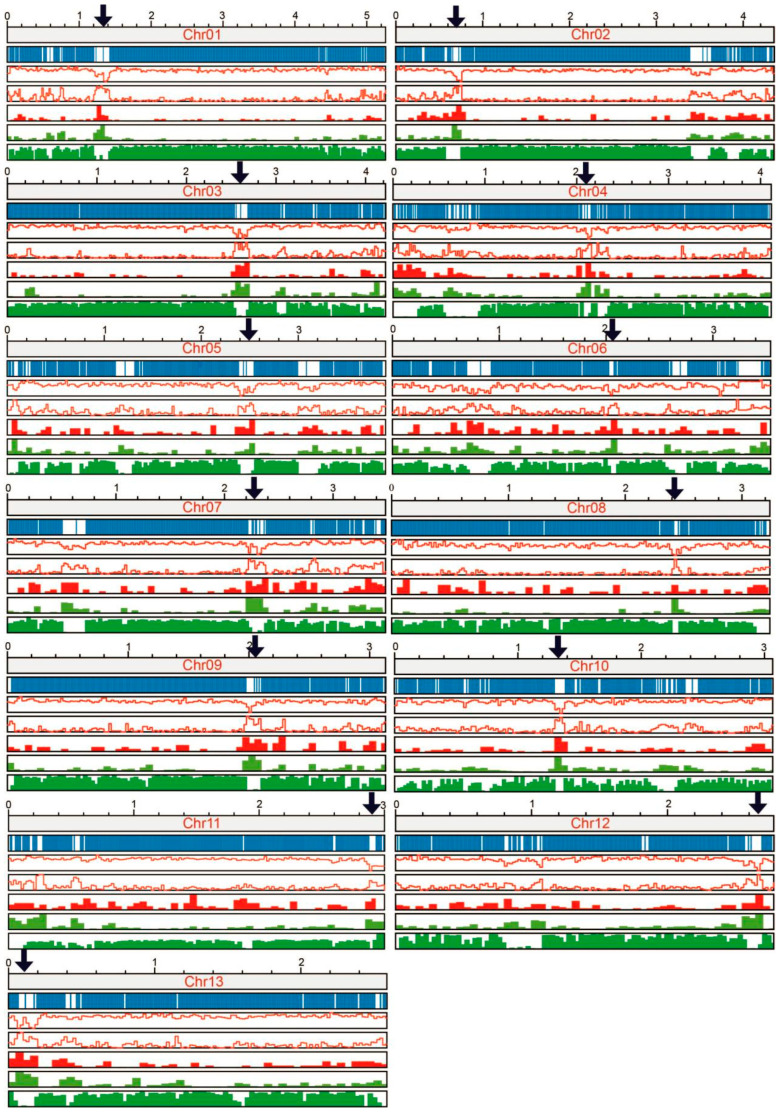
The specific characteristics of centromere regions. The top to the bottom showed the information of chromosome length (Mb), gene position, GC content, repeat sequences, DNA transposon, retrotransposon and read coverage in RNA-seq, respectively. The arrows indicated the predicted centromere region.

The centromere regions of chromosomes were predicted firstly by high-frequency interchromosomal contact analysis from Hi-C heatmap and then verified by the sequence characteristics and OE intensity. For Chr 01 to Chr 10, the regions of high-frequency interchromosomal contacts were clearly visible on the interaction heatmap (Figure 3B), which were predicted as the centromere regions. Then, they were verified by characteristic plotting which were marked by high repeat content, reduced gene density, low GC content and minimal or no transcription levels compared with the other regions of the chromosome (Figure 5). 

Chromosomes can be distinguished as metacentric, submetacentric, subtelocentric, and telocentric according to the relative location of centromeres [71] (pp. 253–254). Following the classic method of Levan et al. [39], the length differences between long and short arms (d value) and arm ratios (r values) were calculated (Table 2). Chrs 03, 04, 06, and 10 have median centromeres and thus were metacentric (d value of 0–2.5 and r value of 1.0–1.7, Table 2); Chrs 01, 05, 07 and 09 were with submedian centromeres and submetacentric (d value of 2.5–5.0 and r value of 1.7–3.0). Chrs 02 and 08 with subterminal centromeres were subtelocentric (d value of 5.0–7.5 and r value of 3.0–7.0).

For Chrs 11, 12 and 13, the regions of high-frequency interchromosomal contacts of the Hi-C heatmap (Figure 3B) were located at the ends of the chromosomes, making it challenging to determine if they were caused by centromeres or telomeres. However, the presence of highly repeated retrotransposon-like sequences and low GC content at the edges of these chromosomes (Figure 5) suggests that the regions exhibiting high-frequency interchromosomal contact are more likely to be of centromeric nature. These chromosomes were likely to be acrocentric.

The predicted centromere regions based on OE intensity of Hi-C data were found to be in good consistency with the prediction based on Hi-C heatmap and sequence characteristics in most chromosomes. However, for chromosomes whose centromeres were located at the edge or had some noise region (low OE intensity), there was a significant discrepancy between the predictions (Chrs 11, 12, 13). This may be due to the limitations of the algorithm employed [37], which might not be well-suited for accurately predicting centromeres located near the edges of chromosomes (Figure S4).

**Figure 6:**
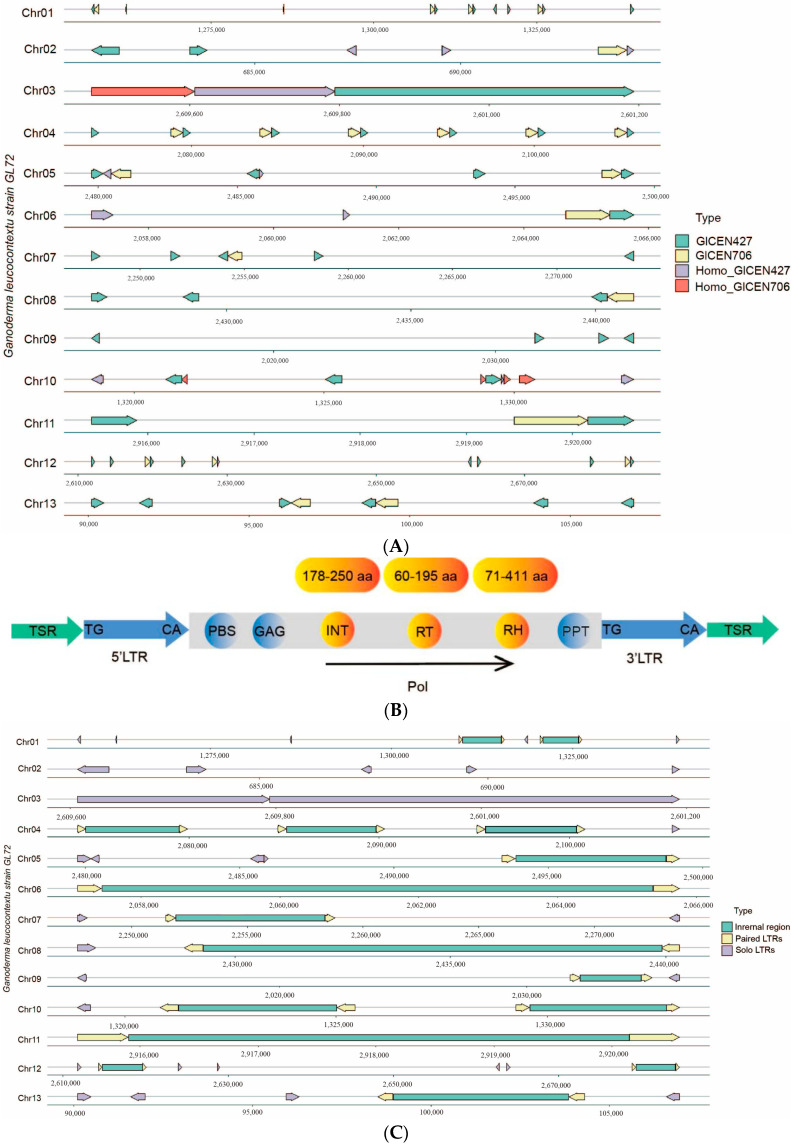
Analysis of conservative sequences in the centromere regions of GL72 genome. (**A**) The distribution of conservative sequences in the centromere regions of GL72 genome. (**B**) The structure of full-length LTR-retrotransposons with both ends of GlCEN427 or homologous sequences. TSR: Target Site Repeat, PBS: Primer Binding Site, GAG: GAG protein, INT: Integrase, RT: Reverse Transcriptase, PPT: Polypurine Tract, pol: polyprotein. (**C**) The distribution of paired and solo LTRs in the centromere regions of GL72 genome. Arrows in different directions indicated the orientation of the LTR.

In most organisms, centromeric sequences consist of short repetitive DNA sequences arranged in tandem and/or transposable elements [72]. Sequence analysis showed that DNA sequence of the predicted centromeric regions of GL72 spanned 30–210 kb (Figure 5). A conservative LTR (long terminal repeats), approximately 427 bp in length, was identified within the centromere regions of all chromosomes (Figure 6A). These LTRs displayed a copy number ranging from 1 to 8 on each chromosome (Table S5). Blast analysis showed that these LTRs were only located at our predicted centromeric regions and had strong conservation among the different chromosomes.

We named it as GlCEN427 and a total of 53 complete and 14 shorter homologous sequences were identified across 13 chromosomes (Table S5). The results obtained from LTR_harvest [46] and LTR_finder [45] revealed that all full-length LTR-retrotransposons with both ends of GlCEN427 or its homologous sequences, including primer binding sites (PBSs) and poly-purine tracts (PPTs), were predicted to be *Copia* LTR-retrotransposons. They encoded four protein domains in the following order: GAG protein, integrase (INT), reverse transcriptase (RT), and ribonuclease H (RH) (Table S6, Figure 6B). Besides, there were some solo LTRs. For Chrs 2 and 3, only solo LTRs were observed. There were 2, 9, and 6 copies of this GlCEN427 sequence in Chrs 11, 12, 13, respectively (Figure 6A), confirming the predicted centromere region of these acrocentric chromosomes. Sequence alignment and phylogenetic analysis revealed that there are some variations of these 53 complete sequences (Figure S5).

Furthermore, another set of conservative sequences, roughly 706 bp in length, was observed in the centromere regions of all the chromosomes with a copy number of 1–6 except Chr 09 (Figure 6A). A total of 22 GlCEN706 sequences and 5 shorter homologous sequences, were found across 13 chromosomes and they always accompany along with GlCEN427. GlCEN706 was also only located at our predicted centromeric regions. There were 1, 3, and 2 copies in Chrs 11, 12, 13, respectively (Figure 6A, Table S5). It was annotated as RH by alignment with the GyDB database [73]. Transcriptomic data suggested that they were not entirely in a silenced state (Table S6).

We tried to blast the high-quality genomes of *G. leucocontextum* strain CCMJ4170 using sequences of GlCEN427 and GlCEN706. It was found that both sequences were also enriched in a certain region of all chromosomes and had multiple copies (Figure S6). Blast against other species in *Ganoderma* with high-quality genomes [9] showed that GlCEN427 sequence only existed in the genome of *G. tsugae* strain CCMJ2475 (Figure S7) and there was no homologous sequence in the genomes of *G. sichuanense* strain CCMJ3025 (Figure S8), *G. multipileum* CCMJ3051 (Figure S9), *G. resinaceum* CCMJ2490 (Figure S10) and *G. sinene* strain CCMJ2497 using the cutoff of e-5. When comparing GlCEN427 sequences with the NCBI reference genome database, matches were only discovered with polyporales fungi such as *D. squalens*, *Rhodofomes roseus*, and *Neoantrodia serialis*. However, matches with the latter two species were limited to a range of 296–427 bp. GlCEN706 was found in almost all the tested genomes of Ganoderma species with different copies or integrity (Figures S6–S10). As for CIGEN706, upon conducting a blastn search in the NCBI genome database with a cutoff of e-5, we noted its occurrence not only in *Polyporaceae* but also various species of Basidiomycota, such as *Schizophyllum commune*, *Mycena indigotica*, *Agaricus bisporus*, and so forth. Notably, it was absent in Ascomycetes.

### 3.4. Gene prediction and Genome-Wide Functional Annotation 

The prediction of TEs and other repetitive DNA sequences identified that these regions comprised approximately 6.62 Mb, covering 14.19% of the genome (Table 3). In detail, TEs and tandem repeats accounted for 11.82% and 0.47%, and a total of 2286 SSRs were identified, covering 0.06% of the genome with a length of 190,641 bp. LTR retrotransposons were the most abundant TE, which covered 8.28% of the genome (Table 3 and Figure 3A).

Multiple tools were used to predict gene structure and function for high accuracy (Table S7). The final integration led to a dataset containing 12,493 protein-coding gene models with an average gene sequence length of 2125.25 bp. For those gene models, the average CDS length was 1433.83 bp, with an average exon number of 6.6 and 217.23 bp. The majority of genes exhibit an even distribution across the chromosomes (Figure 4). 

Based on the comparison of the databases (NR, KEGG, KOG, SwissProt and GO), a total of 11,528 genes were annotated, representing 92.28% of the predicted genes. According to the GO database, the first five GO categories were “metabolic process”, “binding”, “catalytic activity”, “cellular process”, and “single-organism process” (Figure S11). By mapping to the KEGG database, a total of 986 (25.73%) proteins were classified as the category “global and overview maps” (Figure S12). Other highly represented pathways were “signal transduction” with 327 (8.53%) and “transport and catabolism” with 361 (9.42%).

### 3.5. Phylogenomic and Whole-Genome Collinearity Analyses 

**Figure 7:**
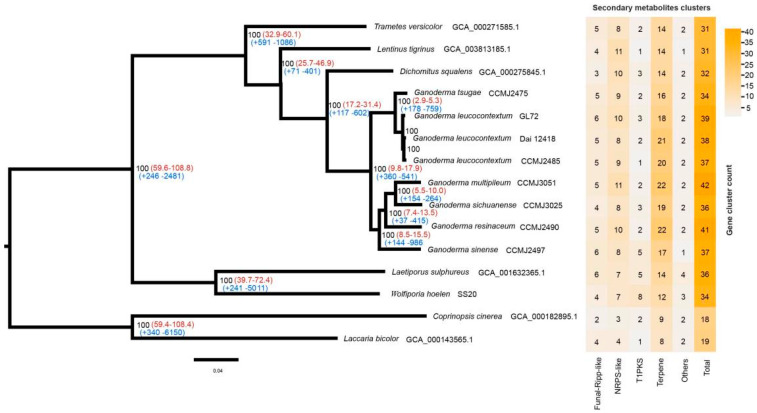
Phylogenomic analysis and secondary metabolite cluster heatmap among 15 fungal genomes. Bootstrap values were indicated in black, divergence time with 95% CI (confidence interval) in red and gene family expansion/contraction in blue.

The whole-genome sequences of 13 species of Polyporales and 2 species of Agaricales were used for phylogenomic analysis (Table S1). Other than *G. leucocontextum*, 5 species of *Ganoderma* that have high-quality genome assembly [9] were chosen, including three species cultivated widely in China, *G. sichuanense*, *G. sinense* and *G. tsugae*. The clustering of proteomes resulted in 2137 single-copy orthologous genes among 15 fungi. A maximum likelihood (ML) phylogeny analysis was performed based on the shared single-copy orthologous genes, which were concatenated into a supermatrix with 754,108 amino acid sites. *G. leucocontextum* was phylogenetically close to *G. tsugae*, as they clustered a group and the other 4 *Ganoderma* species as another group with high bootstrap values (Figure 7). Phylogenomic analysis supported the different species of *G. leucocontextum* and *G. sichuanense*, which were often mixed in the market. 

Using the molecular clock method, the divergence time was estimated using the fossil record of *L. bicolor* and *C. cinerea* as calibration points [54]. It was found that the genus *Ganoderma* diverged from *D. squalens* approximately 17.2–31.4 MYA. *G. leucocontextum* and *G. tsugae* diverged from the other *Ganoderma* species approximately 9.8–17.9 MYA. *G. tsugae* diverged from *G. leucocontextum* approximately 2.9–5.3 MYA (Figure 7). 

Furthermore, there were 360 expanded and 541 contracted gene families between two branches of the genus *Ganoderma*. KEGG enrichment analysis demonstrated that the expanded gene families were involved in a variety of vital biological processes, including purine and nucleotide metabolism, cofactors and vitamins metabolism, DNA replication, and more. In contrast, the contracted gene families were primarily associated with processes such as cyanoamino acid metabolism, amino sugar and nucleotide sugar metabolism, carbohydrate metabolism, starch and sucrose metabolism, biosynthesis of various plant secondary metabolites, and cytochrome P450 activity and so on (Table S8).

There were 71 unique gene families in GL72. KEGG enrichment analysis revealed that they were mainly related to terpenoid backbone biosynthesis, amino sugar and nucleotide sugar metabolism and so on (Figure S13). Using chromosome information, we conducted whole-genome conservation synteny analysis for *G. tsugae*, *G. sichuanense* and GL72. Despite variations in intra-chromosome structure, such as some insertions, deletions, and inversions, the whole-genome sequences of the *Ganoderma* species showed good synteny (Figure S14).

### 3.6. Prediction of Secondary Metabolite Clusters 

Based on the high-quality genome of *Ganoderma* species, secondary metabolites clusters were identified by antiSMASH v 7.0.0. In the 10 *Ganoderma* species studied, a total of 34–47 clusters, which includes 16–26 terpene clusters, were predicted for each species (Table S9). These findings confirm the genetic foundation underlying the production of abundant terpenoids in the fruiting bodies of *Ganoderma* species. There were 39 predicted clusters, among which 18 were terpene clusters for *G. leucocontextum* strain GL72. These clusters distributed on chromosome unevenly. There were 5 or over 5 clusters on Chrs 02, 08 and 10, and no predicted on Chr 4 and Chr 11 (Figure 4). 

For *G. leucocontextum* strain GL72, two terpene clusters (cluster 3.1 and 13.3) were predicted for (+)-δ-cadinol, terpene cluster 5.2 for clavaric acid, NRPS clusters (cluster 3.2) for basidioferrin with 100% of similarity. Clavaric acid has proved to be useful in cancer therapy as a selective inhibitor of Ras farnesyl transferase, competing reversibly with the Ras-peptide substrate without affecting the biosynthesis of isoprenoids [74,75]. Other than *G. leucocontextum*, the cluster for clavaric acid was also predicted in the three cultivated *Ganoderma* species, *G*. *tsugae*, *G*. *sichuanense* and *G*. *sinense*. Plant extracts containing cadinene-type sesquiterpenes (e.g., δ-cadinol) were shown to have anti-microbial, anti-fungal and anti-inflammatory activities. To date only four terpene synthases responsible for the synthesis of δ-cadinol have been identified, BvCS from *Boreostereum vibrans* [76], GME3638 from *Lignosus rhinocerotis* [77], Copu5 and Copu9 from *Coniophora puteana* [78]. Two clusters for (+)-δ-cadinol for both *G. leucocontextum* and *G. sichuanense* were predicted and showed high similarity with Copu5 and Copu9 from *C. puteana* (Figure S15). These findings provide genetic evidence supporting the high pharmacodynamic activity of the fruiting bodies of *G. leucocontextum* and will be beneficial for the discovery of novel active substances and elucidation of the metabolic pathway. 

Compared with the widely cultivated species *G. sichuanense*, *G. tsugae* and *G. sinene,* 1 cluster for terpene (core gene LG02.1231) was found to be unique in *G. leucocontextum* (Figure 4, Table S10).

## 4. Discussion

A complete T2T finished genome has been the long pursuit of genomic research [40]. Here, we reported the first gap-free T2T genome of Tibetan medicinal mushroom *G. leucocontextum* using a combination of multiple sequencing, including NGS, PacBio HiFi and Hi-C techniques. The centromeres and telomeres of all 13 chromosomes were successfully assembled. A variety of assessment strategies, including BUSCO, Merquery, high mapping rate and uniform coverage, demonstrated the integrity and accuracy of this genome assembly. The sequence analysis revealed the first *Copia* centromeric retrotransposon in macro-fungi genome. Prediction of secondary metabolic clusters confirmed the capability of this fungi to produce a substantial quantity of metabolites. This gap-free T2T genome of *G. leucocontextum* will be contribute to the genomic ‘dark matter’ elucidation, new natural product discovery, metabolic engineering, and so on.

Three distinct genomes for *G. leucocontextum* have been published previously [7,8,9]. However, in each of these instances, certain segments of the genome have remained incompletely assembled. In this study, we successfully achieved a chromosome-level genome assembly for *G. leucocontextum* which contains 13 full-length nuclear chromosomes. By manually correcting the regions with localized coverage anomalies and completing the missing telomere of Chr 11, we ultimately obtained a T2T gap-free assembly, the termini of which all display typical telomeric sequences (i.e., TTAGGG at 3′-termini and the reverse complement CCCTAA at 5′-termini). We have tried to confirm the results using pulsed-field gel electrophoresis, but failed after numerous attempts. However, no noticeable noise was observed outside the diagonal from the Hi-C heatmap (Figure 3B), which can confirm the correctness of the assembly. 13 chromosomes of *G. leucocontextum* were also reported in the previous study [9].

The extensive tandem duplication within the rDNA sequences posed a significant challenge for achieving a comprehensive assembly. Through calculations of total length and coverage of rDNA sequence following the method of Chen et al. [40], it was estimated that the number of rDNA copies in the *G. leucocontextum* genome is 72. This estimation was consistent with the previously report on fungal rDNA copies, which had a mean of 98 and a median of 82 copies [79]. To accurately assemble the rDNA and intergenic spacer region sequences, we extracted all reads associated with these regions, and made efforts to manually correct and connect contigs using variations identified within the intergenic spacer of rDNA. However, due to the limited read length of HiFi reads and the challenges encountered in assembling repetitive sequences, precise completion of the rDNA sequences proved challenging. The rDNA sequences at the 3′ terminal of Chr 11 is the flaw of our genome assembly. Further experimental validation or utilization of longer reads may be necessary to address this limitation in future iterations.

Although the role of the centromere is conserved throughout evolution, the DNA sequences associated with centromere regions are highly divergent among species. In most eukaryotes, centromeric DNAs are typically composed of highly repeated retrotransposon-like and satellite sequences [80,81]. In the case of *Drechmeria coniospora* [82], the centromere region demonstrates heightened repeat content, diminished gene density, and lowered GC content. A different pattern emerges in the centromere region of *Tricholoma matsutake* [83], which were characterized by GC-rich, LINEs-rich, and devoid of LTRs. These findings suggested substantial differences in genome structure between centromeric regions and other sections of the chromosomes, and great variation among different species. In this study, regions characterized by high repeat content, decreased gene density, low GC content, highest Hi-C interaction intensity, and minimal or no transcription levels at a specific position across all chromosomes, may represent potential centromeres (Figure 5). The possible regions of the centromeres were spanned 30–210 kb. It was reported that the centromeric DNA sequence was spanned 4–4.5 kbp in the yeast *Candida lusitaniae, Candida albicans* 3–5 kb, *Schizosaccharomyces pombe* 35–110 kb determined by ChIP-sequencing [84]. We have also tried two software tools, quarTeT [85] and TBtools [35], which have been reported to predict the centromere regions in *Actinidia chinensis*, *Arabidopsis thaliana*, and *Oryza sativa*, however, the accurate prediction of chromosomal centromeric regions remained elusive. It could be attributed to these two software tools relying on tandem repeat searches, making them more suitable for analyzing the genome with complex and highly repetitive sequences. 

*Ty3/Gypsy* retrotransposon sequences have gained evolutionary advantage in the centromeres of most species, especially in plant [86]. For instance, in maize, *Gypsy* elements are notably abundant in centromeric regions and show a negative correlation with gene distribution [87]. However, in some plants such as *Brassica rapa* [88], *Triticum aestivum* [89] and *Nelumbo nucifera* [90], centromeric regions have been observed to be primarily associated with *Ty1/Copia* retrotransposons. This research has revealed a distinct class of *Copia* LTR-RT elements, named GlCEN427, which is found exclusively in centromeric regions of the genome. The absence of GlCEN427 sequences in the high-quality genomes of *G. sichuanense* and *G. sinene* [9] but their presence in polyporales fungi such as *D. squalens*, *R. roseus*, and *N. serialis*, suggests that the GlCEN427 sequences in centromeric regions have been vertically inherited from a common ancestor, and undergone loss and gain repetitively during evolution. Some LTR-RTs of GlCEN427 bears all features essential for retrotransposon, which is composed of two nearly identical LTR sequences, flanked by target site repeats (TSRs), internal region containing open reading frame, GAG, INT, RT, RH (Figure 6B). It was reported that the most distinctive structural feature of a centromeric retrotransposon is the presence of an integrase chromodomain, which is widely assumed to ensure correct targeting to the centromeric region [91]. Another conserved sequence named GlCEN706 within the centromeric regions was annotated as RH. GlCEN706 has been identified in the majority of *Ganoderma* species and consistently coexists with GlCEN427 in both *G. leucocontextum* and G. *tsugae*. Further in-depth studies were needed to elucidate the specific functions of these *Copia* LTR-RTs. 

Telomeres are repetitive sequences located at the ends of chromosomes, and play a crucial role in cell survival by protecting chromosome ends and regulating their movement during both mitosis and meiosis [92,93]. It has been reported that highly conserved sequences encoding RecQ helicases are present at several fungal chromosomal ends, located very close to the telomere repeat [94]. We identified genes in our genome that potentially encode RecQ helicases based on the hidden markov model profile for RecQ helicase (PTHR13710) using hmmsearch 3.3.2 [95] with a cutoff of e-5, and it was found that genes encoding RecQ helicases were present at near some telomeres, including Chrs 04, 06, 08, 12 (Table S11).

Based on this T2T genome assembly of *G. leucocontextum* and the relative high-quality genome assembly of other species within *Ganoderma* [9], a phylogenomic analysis was performed and the relation among species of *Ganoderma* genus was focused. *G. leucocontextum* was confirmed to be phylogenetically close to *G. tsugae* (Figure 7), which supported the results of previous report with ITS sequences [8] and phylogenomic analysis [9]. The divergence times inference with *C. cinerea* and *L. bicolor* as calibration points [54] indicated that the genus *Ganoderma* diverged from *D. squalens* approximately 17.2–31.4 MYA, which is consistent well with a *Ganodermites libycus* fossil record dating back possibly to 18–19 MYA [96]. This result provided genomic clues to the potential emergence of *Ganoderma* species in the Miocene epoch (23.03–5.33 MYA). Miocene epoch is a time of warmer global climates than those in the preceding Oligocene or the following Pliocene [97]. *G. leucocontextum* was found to have a lower optimal growth temperature compared to other cultivated *Ganoderma* species such as *G. Sichuanese*. The divergence of *G. tsugae* from *G. leucocontextum*, estimated to have occurred approximately 2.9–5.3 MYA during the Pliocene period (5.332 to 2.588 Ma), appears to be reasonable.

Due to its rich metabolites, *G. leucocontextum* possesses higher medicinal and economic value than ordinary *Ganoderma* species [98]. Out of the 37 secondary metabolite gene clusters, 20 were predicted to be involved in terpene biosynthesis. This result is consistent with the discovery of numerous triterpenes in previous studies [2,3,4,6]. This high-quality genome will be contributed to the new compound discovery and metabolic engineering of secondary metabolite production of this Tibetan medicinal mushroom.

## Figures and Tables

**Table 1:** Global statistics for the genome of *Ganoderma leucocontextum* strain GL72 and the previously published strains.

Assembly	GL72	HMGIMI 160015	Dai 12418	CCMJ 4170
Sequencing technology	MGISEQ2000, PacBio, Hi-C	Illumina, Nanopore	PacBio	PacBio, Hi-C
Assembly level	Chromosome	Contig	Contig	Chromosome
Assembly length (Mb)	46.69	50.05	60.34	47.42
Number of contigs (gaps)	13 (0)	58 (0)	843 (195)	13 (2800)
Number of telomeres	26	0	0	14
Contig N50 (bp)	3,539,945	3,064,430	205,166	3,391,645
Contig N90 (bp)	2,781,080	481,045	32,539	2,727,109
Repeat content (%)	14.19	12.64	/	16.51
GC content (%)	56.24	55.85	55.95	56.1
Complete BUSCOs (%)	99.7	96.55	99.8	99.2
Protein-coding prediction	12,493	13,390	16,952	12,606
Average gene length (bp)	2125.25	2209	1846	2709
Reference	This study	[7]	[8]	[9]

**Table 2:** The predicted centromere region.

ID	Length (Mb)	s	l	d	r	R	Type
Chr 01	5.26	2.5	7.5	4.99	3	0.226	submetacentric chromosome
Chr 02	4.35	1.55	8.45	6.9	5.45	0.756	subtelocentric chromosome
Chr 03	4.22	3.81	6.19	2.38	1.62	0.576	metacentric chromosome
Chr 04	4.12	4.92	5.08	0.17	1.03	0.416	metacentric chromosome
Chr 05	3.9	3.62	6.38	2.75	1.76	0.756	submetacentric chromosome
Chr 06	3.54	4.18	5.82	1.64	1.39	0.627	metacentric chromosome
Chr 07	3.48	3.46	6.54	3.08	1.89	0.371	submetacentric chromosome
Chr 08	3.24	2.49	7.51	5.02	3.02	0.715	subtelocentric chromosome
Chr 09	3.13	3.5	6.5	2.99	1.85	0.579	submetacentric chromosome
Chr 10	3.07	4.29	5.71	1.41	1.33	0.464	metacentric chromosome
Chr 11	3.00	0.33	9.67	9.34	29.3	0.691	acrocentric chromosome
Chr 12	2.78	0.25	9.75	9.5	39	0.683	acrocentric chromosome
Chr 13	2.59	0.39	9.61	9.22	24.64	0.567	acrocentric chromosome

l and s: the length of the long arm and short arm; d: length difference between long and short arm, d = l − s; r: ratio r = l/s; R: centromere ratio of chromosomes predicted by Hi-C interaction intensity.

**Table 3:** Classification of the repeat sequences in the genome of *Ganoderma leucocontextum* GL72.

Class	Order	Super Family	Number of Elements	Length of Sequence (bp)	Percentage of Sequence (%)
Class I			5042	4,367,328	9.35
	LINE		809	485,316	1.04
		Penelope	57	99,793	0.21
		Unknown	701	344,587	0.74
		Other	51	40,936	0.09
	LTR		4128	3,866,724	8.28
		Unknown	2345	2,040,116	4.37
		Gypsy	1376	1,518,422	3.25
		Copia	363	244,159	0.52
		Pao	32	60,423	0.13
		Other	12	3604	0.01
	SINE		105	15,288	0.03
		Other	105	15,288	0.03
Class II			2397	1,150,196	2.46
	DNA		1225	701,114	1.5
		Unknown	1022	544,336	1.17
		hAT-Ac	101	108,927	0.23
		Other	102	47,851	0.1
	MITE		1125	363,180	0.78
		Unknown	1125	363,180	0.78
	RC		47	85,902	0.18
		Helitron	47	85,902	0.18
Total TEs			7439	5,517,524	11.82
Tandem Repeats			4790	218,982	0.47
	SSR		2286	28,341	0.06
	Tandem repeat		2504	190,641	0.41
Simple repeats			103	11,754	0.03
Unknown			1721	873,658	1.87
Low complexity			9	1405	0
Other			3	303	0
Total Repeats			14,065	6,623,626	14.19

## Data Availability

The raw sequences of PacBio long-read were submitted to NCBI SRA (http://www.ncbi.nlm.nih.gov/sra (accessed on 7 October 2023)) under BioProject accession numbers PRJNA1025122.

## Supplementary Materials

The following supporting information can be downloaded at: https://www.mdpi.com/article/10.3390/jof10010015/s1, Figure S1. Heterozygosity analysis; Figure S2. Mitochondrial genome of *Ganoderma leucocontextum* strain GL72. Figure S3. Regions with localized coverage anomalies; Figure S4. Predicted Centromere regions by OE intensity of Hi-C data; Figure S5. Phylogenetic analysis of 53 complete GlCEN427 sequences; Figure S6. The distribution of GlCEN427, GlCEN706 and their homologous sequences in the genome of *Ganoderma leucocontextum* strain CCMJ4170; Figure S7. The distribution of GlCEN427, GlCEN706 and their homologous sequences in the genome of *Ganoderma tsugae* strain CCMJ2475; Figure S8. The distribution of GlCEN427, GlCEN706 and their homologous sequences in the genome of *Ganoderma sichuanense* strain CCMJ3025; Figure S9. The distribution of GlCEN427, GlCEN706 and their homologous sequences in the genome of *Ganoderma multipileum* CCMJ3051; Figure S10. The distribution of GlCEN427, GlCEN706 and their homologous sequences in the genome of *Ganoderma resinaceum* CCMJ2490; Figure S11. Gene annotation according to the GO database; Figure S12. Gene annotation according to the KEGG database; Figure S13. KEGG enrichment analysis of unique gene families in GL72; Figure S14. whole-genome conservation synteny analysis for *Ganoderma tsugae*, *Ganoderma sichuanense* and GL72; Figure S15. the alignment results of clusters for (+)-δ-cadinol for *Coniophora puteana*, *Ganoderma sichuanense*, *Ganoderm sinene* and GL72; Table S1. Genomes for phylogenomic tree; Table S2. The chromosome length results; Table S3. Genome quality assessment using BUSCO; Table S4. N counts of the genome of Ganoderma leucocontextum CCMJ4170; Table S5. Repeat counts in centromere regions; Table S6. Annotation of full-length LTR-retrotransposons with both ends of GlCEN427 or homologous sequences; Table S7. Prediction results of gene structure; Table S8. KEGG enrichment analysis of expanded andcontracted gene families between two branches of the Ganoderma genus; Table S9. Gene clusters numble found in antiSMASH; Table S10. Unique clusters in GL72; Table S11. Gene family of RecQ helicases.
