# Supplementary material for: Telomere-to-Telomere Genome Assembly of Tibetan Medicinal Mushroom Ganoderma leucocontextum and the First Copia Centromeric Retrotransposon in Macro-Fungi Genome
Source: J Fungi (Basel). 2023 Dec 27;10(1):15. doi: 10.3390/jof10010015 (PMC10817607; doi:10.3390/jof10010015)
Supplement: Supplementary file 1 [file jof-10-00015-s001.zip › jof-2778787-supplementary.pdf]

Telomere-to-telomere genome assembly of Tibetan medicinal mushroom *Ganoderma leucocontextum* and the first *Copia* centromeric retrotransposon in macro-fungi Genome

Miao Wang[1, 2], Guoliang Meng[1, 2], Ying Yang[1, 2], Xiaofang Wang[1], Rong Xie[3] and Caihong Dong[1*]

[1] *State Key Laboratory of Mycology, Institute of Microbiology, Chinese Academy of Sciences, Beijing, 100101, China*
[2] *University of Chinese Academy of Sciences, Beijing, 100049, China*
[3] *Institute of VegeSupplementary Table ciences, Tibet Academy of Agricultural and Animal Husbandry Sciences, Lhasa, 850000, China*

Miao Wang, email: mwang2136@gmail.com; Guoliang Meng, email: 18706387822@163.com; Ying Yang, email: yangy@im.ac.cn; Xiaofang Wang, email: wang227529@163.com; Rong Xie, email: xr-20082004@163.com

* Corresponding author
Postal address: No.1 Beichen West Road, Chaoyang District Beijing,100101, P.R. China
Telephone numbers: 86-10-64806193
E-mail addresses: dongch@im.ac.cn (CH Dong)

SUPPORTING INFORMATION

Figure S1

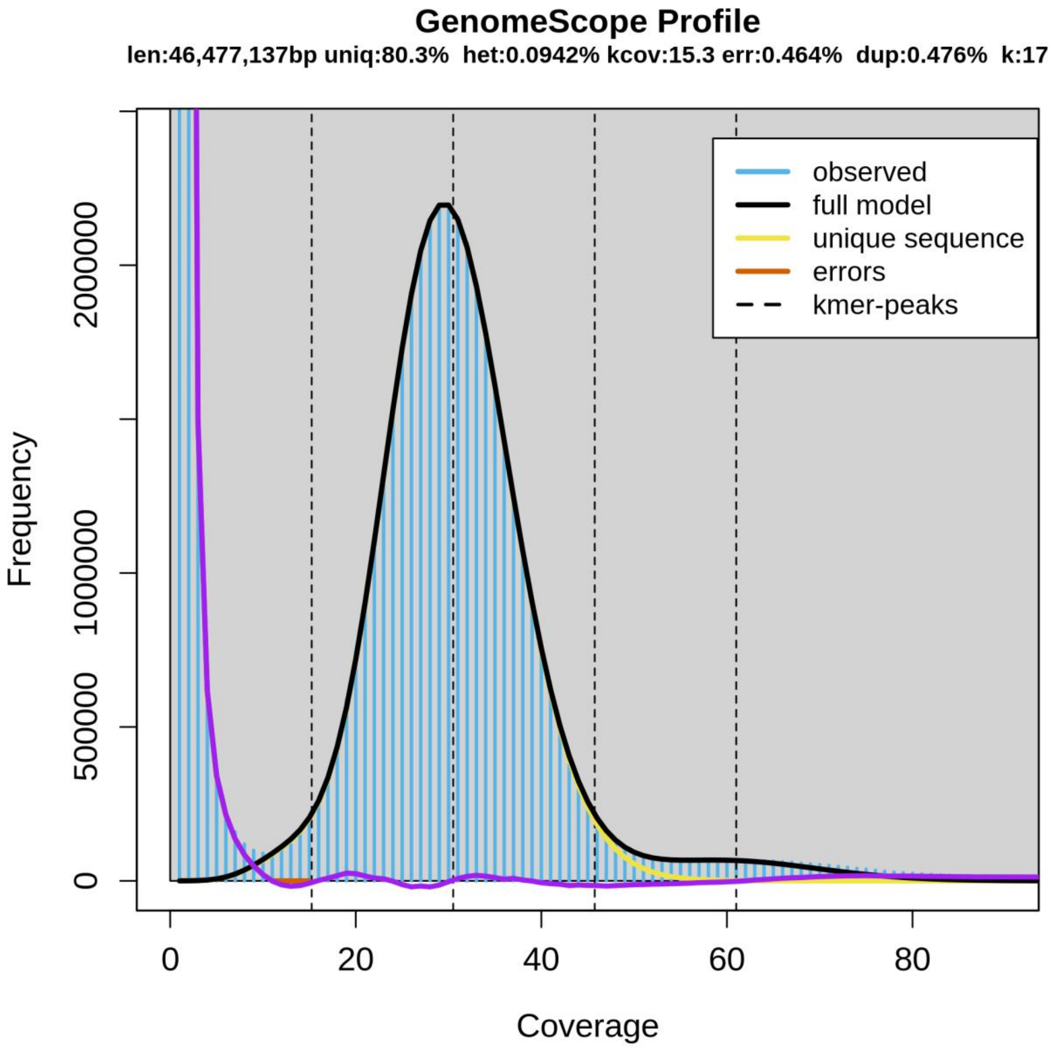

Figure S2

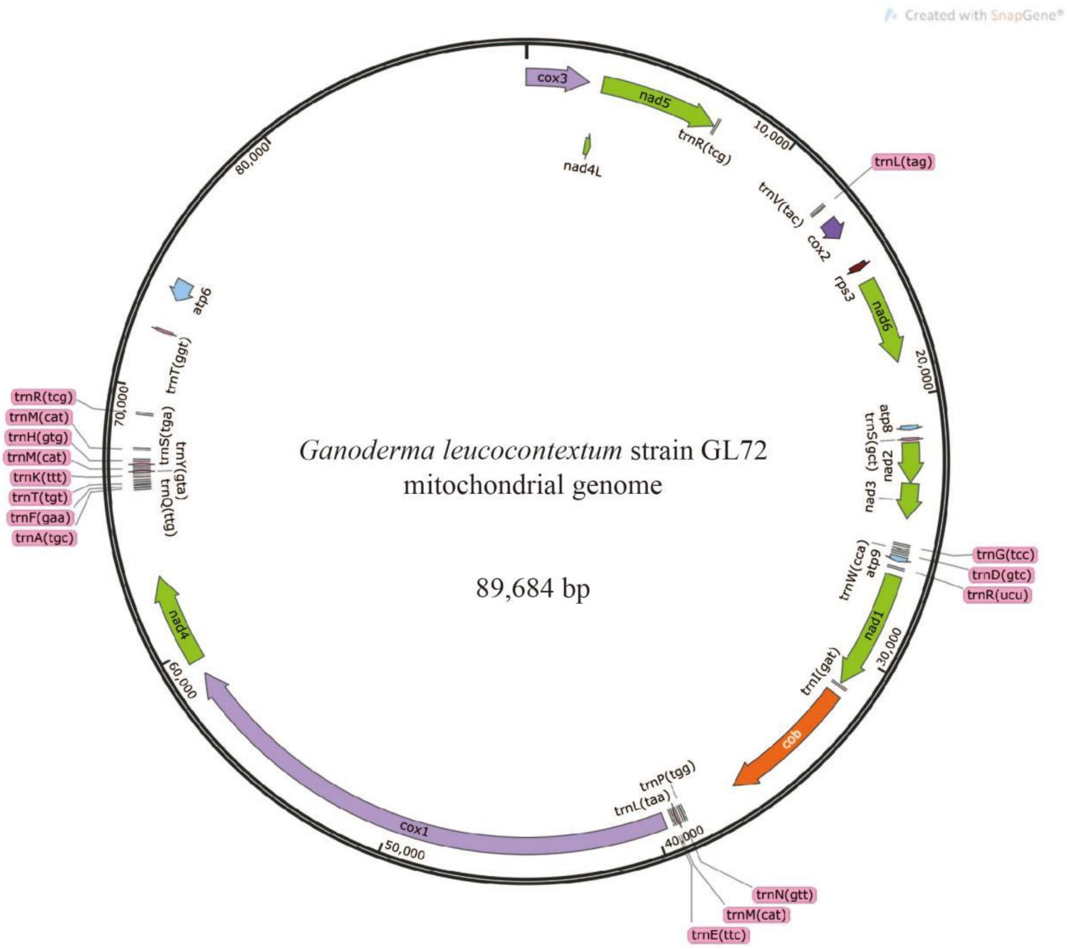
Created with SnapGene®
Ganoderma leucocontextum strain GL72
mitochondrial genome
89,684 bp
cox3
nad5
trnR(tcg)
nad4L
10,000
trnL(tag)
trnV(tac)
cox2
rps3
nad6
20,000
atp8
trnS(gct)
nad2
nad3
trnG(tcc)
trnD(gtc)
trnR(ucu)
trnW(cca)
atp9
nad1
30,000
trnI(gat)
cob
trnP(tgg)
trnL(taa)
40,000
trnN(gtt)
trnM(cat)
trnE(ttc)
cox1
50,000
60,000
nad4
trnR(tcg)
trnM(cat)
trnH(gtg)
trnM(cat)
trnK(ttt)
trnT(tgt)
trnF(gaa)
trnA(tgc)
trnS(tga)
trnY(gta)
trnQ(ttg)
70,000
trnT(ggt)
atp6
80,000

Figure S3

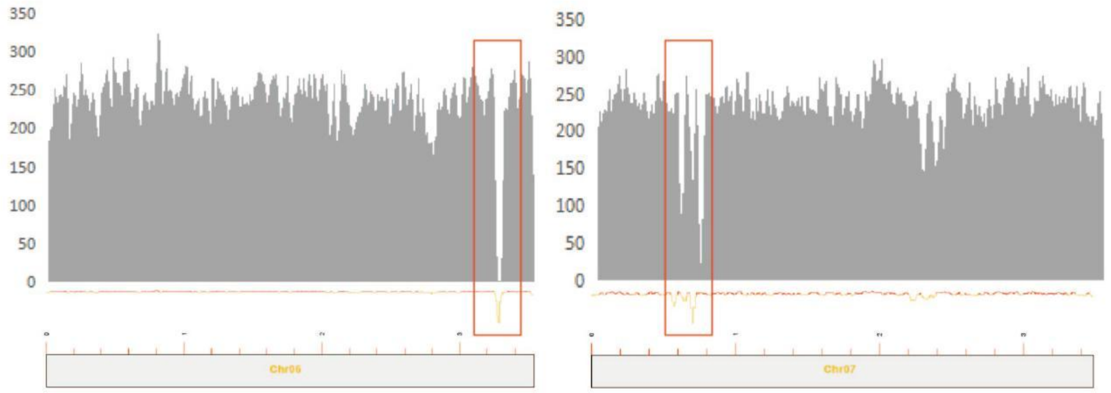
350
300
250
200
150
100
50
0
Chr06
350
300
250
200
150
100
50
0
Chr07

Figure S4

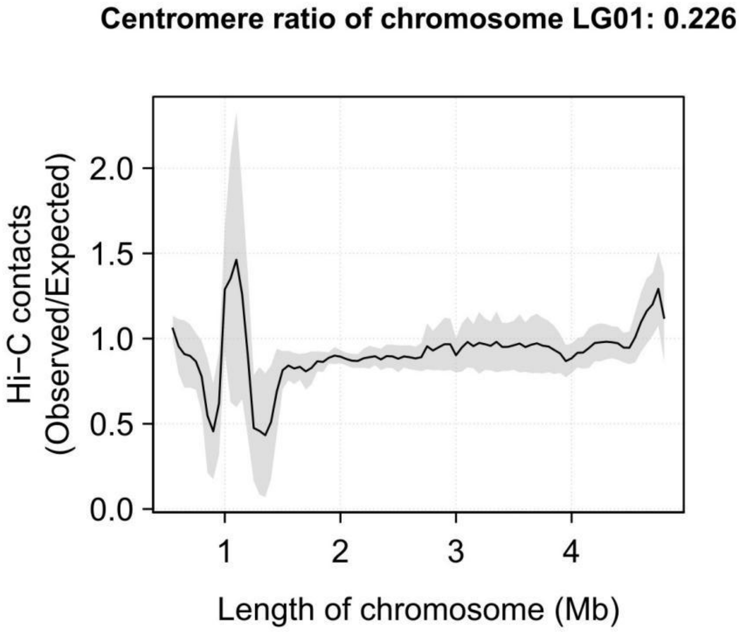
Centromere ratio of chromosome LG01: 0.226
Hi−C contacts (Observed/Expected)
0.0
0.5
1.0
1.5
2.0
1
2
3
4
Length of chromosome (Mb)

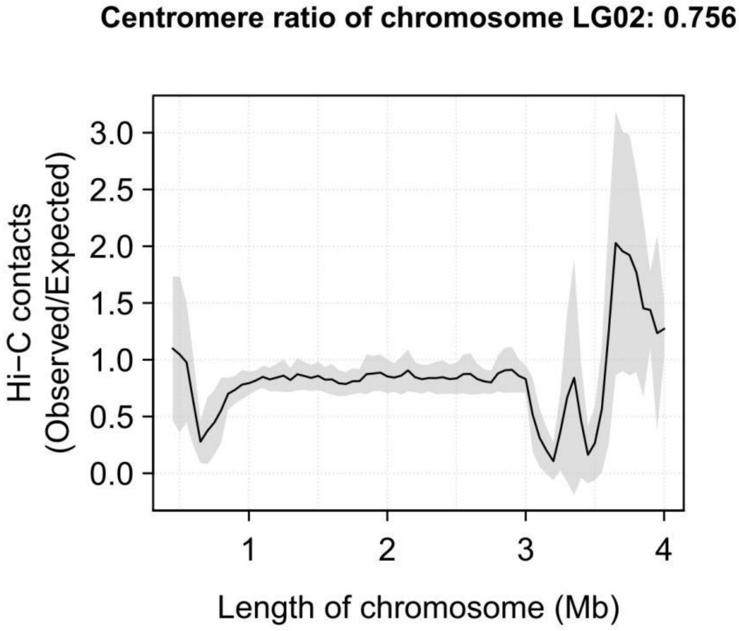
Centromere ratio of chromosome LG02: 0.756
Hi−C contacts (Observed/Expected)
0.0
0.5
1.0
1.5
2.0
2.5
3.0
1
2
3
4
Length of chromosome (Mb)

Centromere ratio of chromosome LG03: 0.576

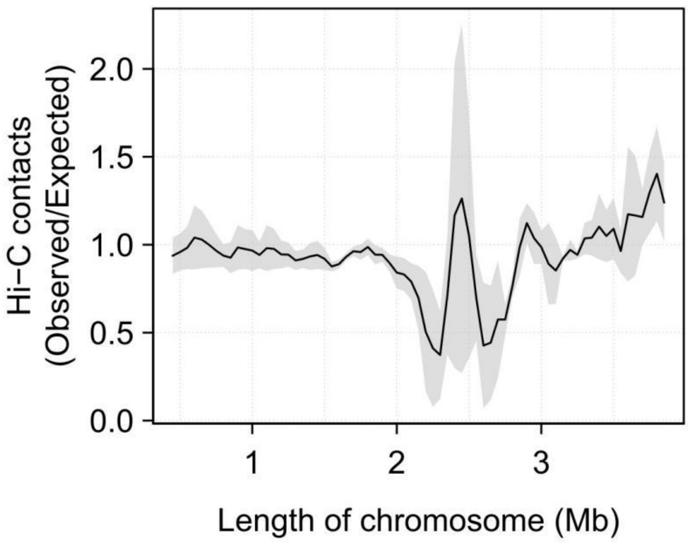

Centromere ratio of chromosome LG04: 0.416

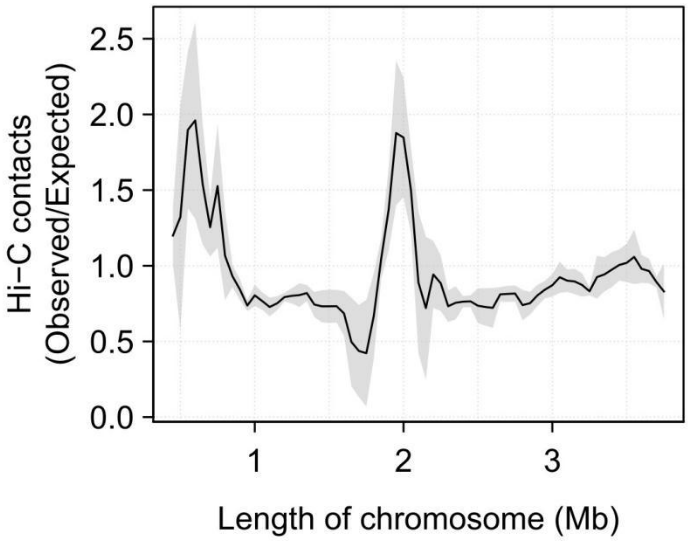

Centromere ratio of chromosome LG05: 0.756

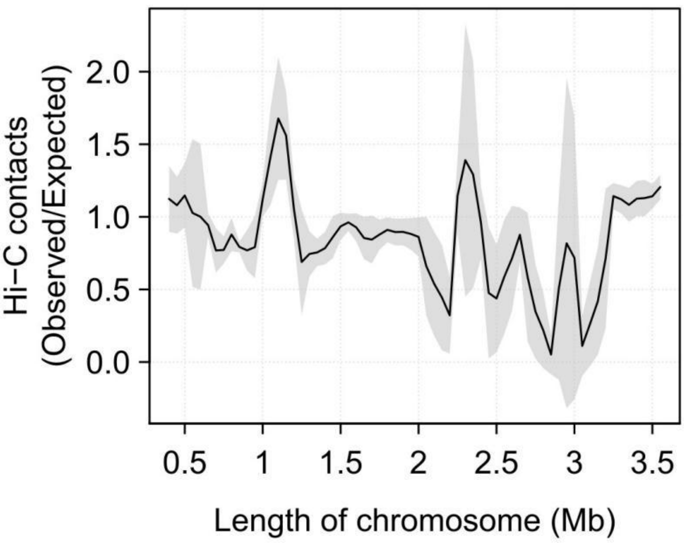

Centromere ratio of chromosome LG06: 0.627

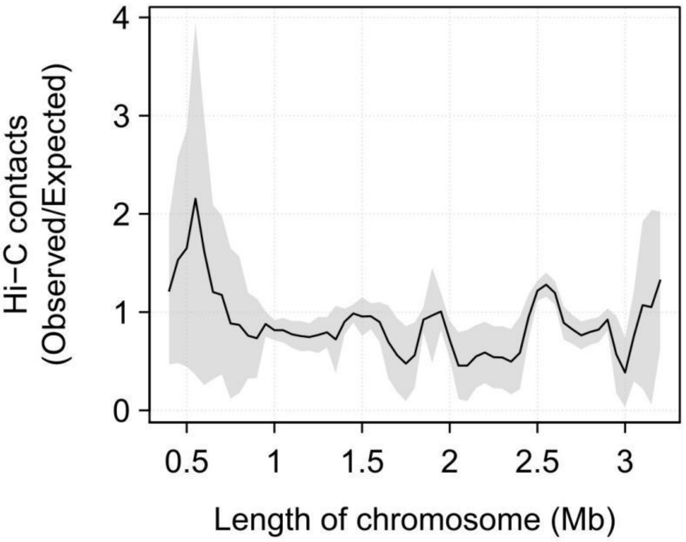

Centromere ratio of chromosome LG07: 0.371

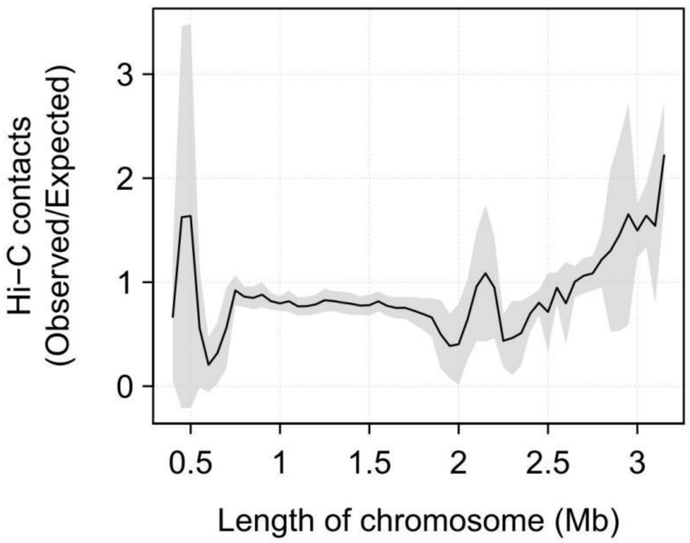

Centromere ratio of chromosome LG08: 0.715

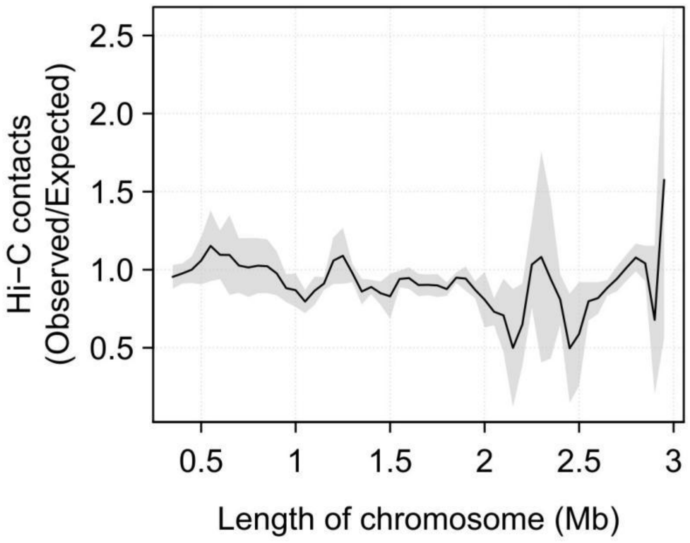

Centromere ratio of chromosome LG09: 0.579

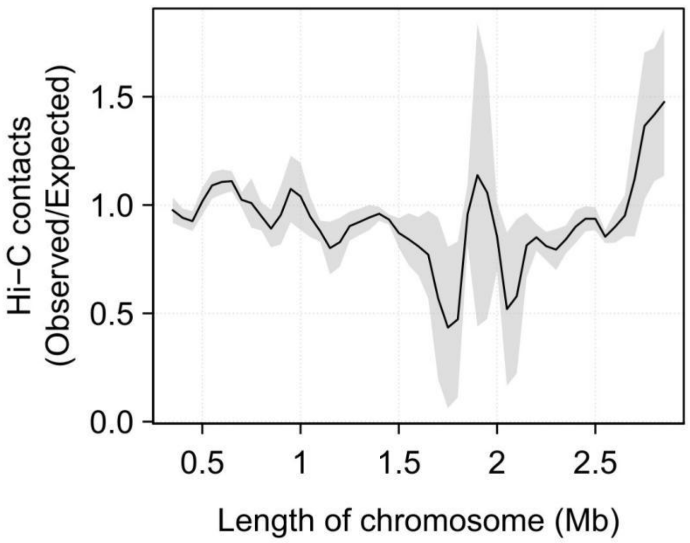

Centromere ratio of chromosome LG10: 0.464

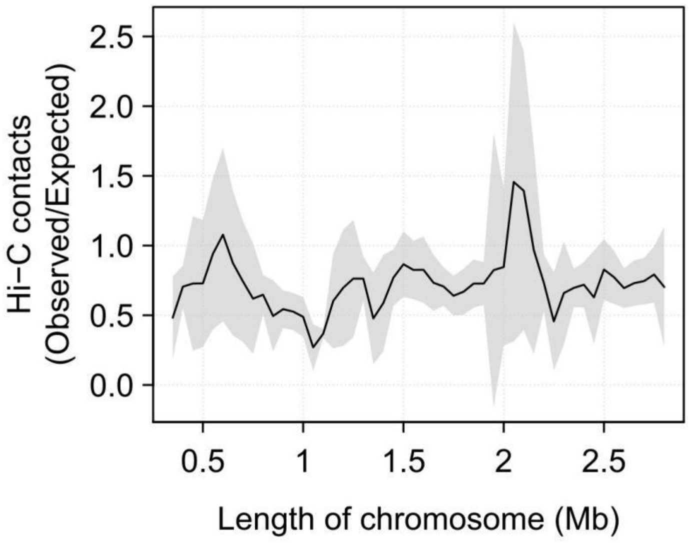

Centromere ratio of chromosome LG11: 0.691

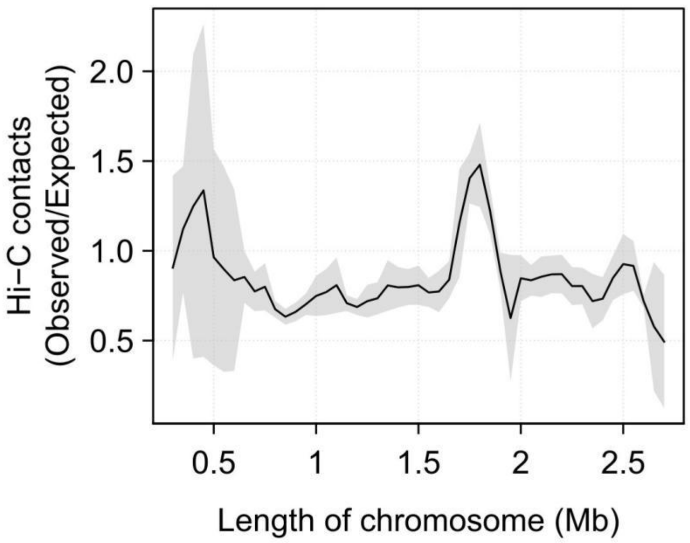

Centromere ratio of chromosome LG12: 0.683

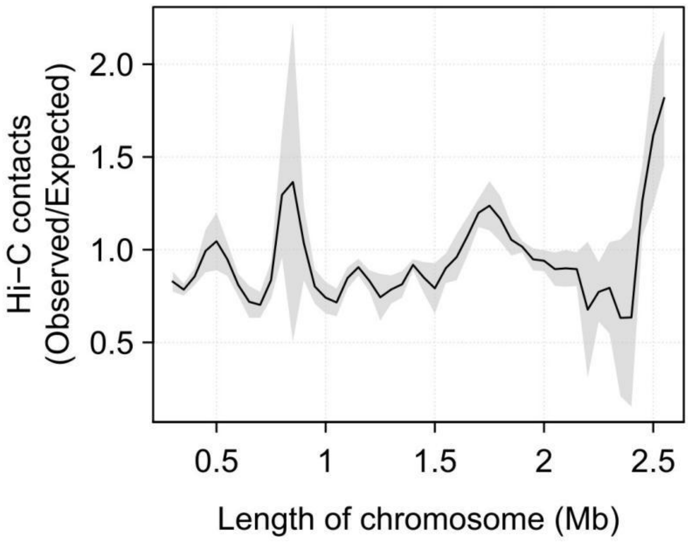

Centromere ratio of chromosome LG13: 0.567

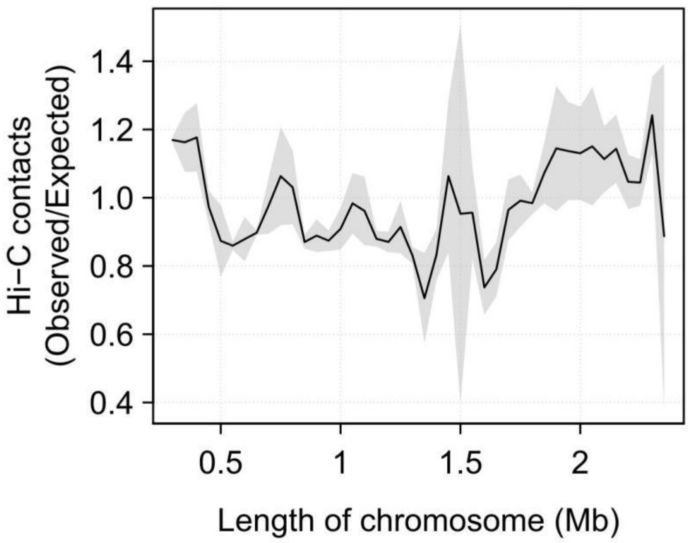

Figure S5

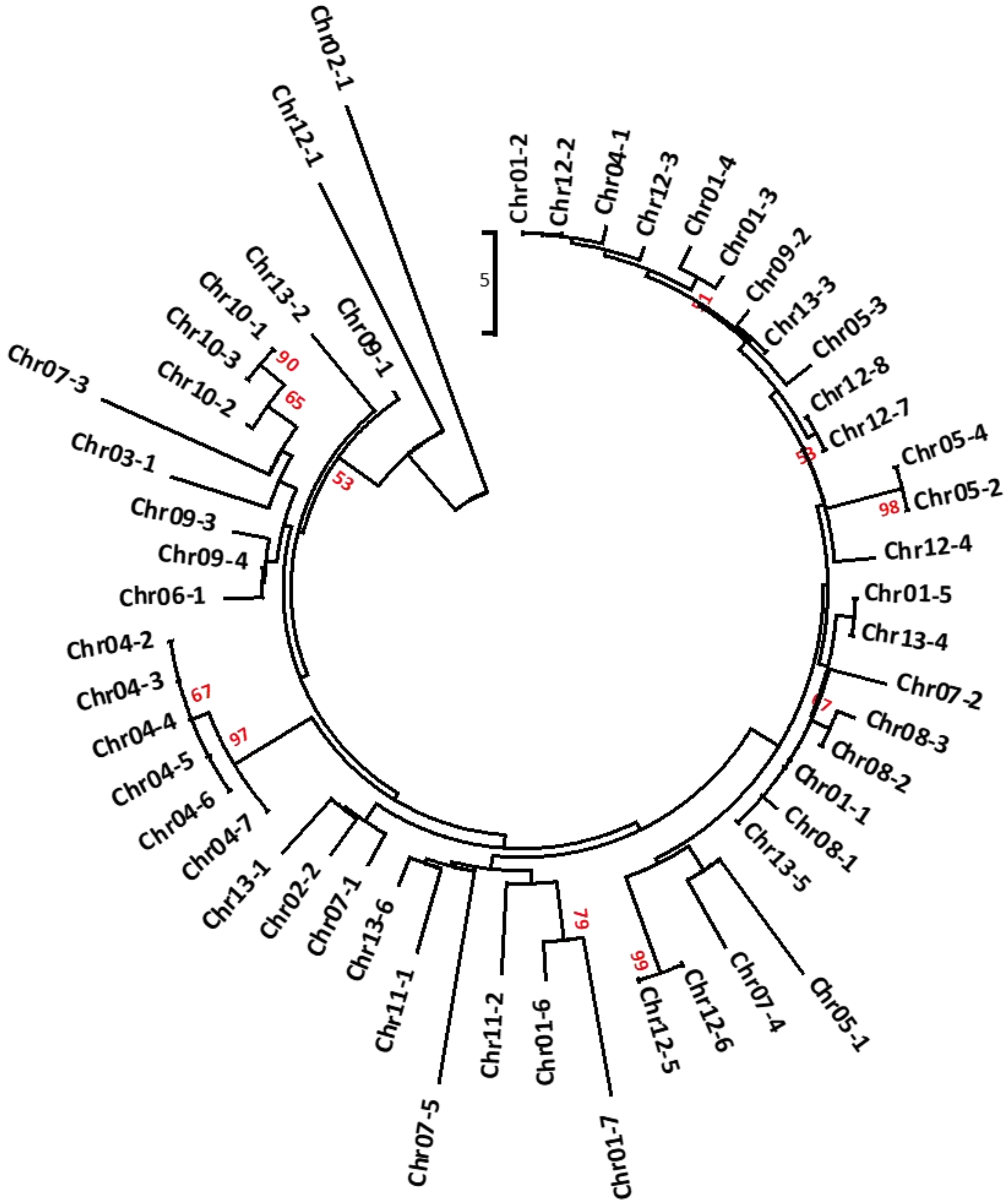

5
Chr02-1
Chr12-1
Chr13-2
Chr09-1
Chr10-1
Chr10-3
Chr10-2
Chr07-3
Chr03-1
Chr09-3
Chr09-4
Chr06-1
Chr04-2
Chr04-3
Chr04-4
Chr04-5
Chr04-6
Chr04-7
Chr13-1
Chr02-2
Chr07-1
Chr13-6
Chr11-1
Chr07-5
Chr11-2
Chr01-6
Chr01-7
Chr12-5
Chr12-6
Chr07-4
Chr05-1
Chr13-5
Chr08-1
Chr01-1
Chr08-2
Chr08-3
Chr07-2
Chr13-4
Chr01-5
Chr12-4
Chr05-2
Chr05-4
Chr12-7
Chr12-8
Chr05-3
Chr13-3
Chr09-2
Chr01-3
Chr01-4
Chr12-3
Chr04-1
Chr12-2
Chr01-2
90
65
53
67
97
79
99
98

Figure S6

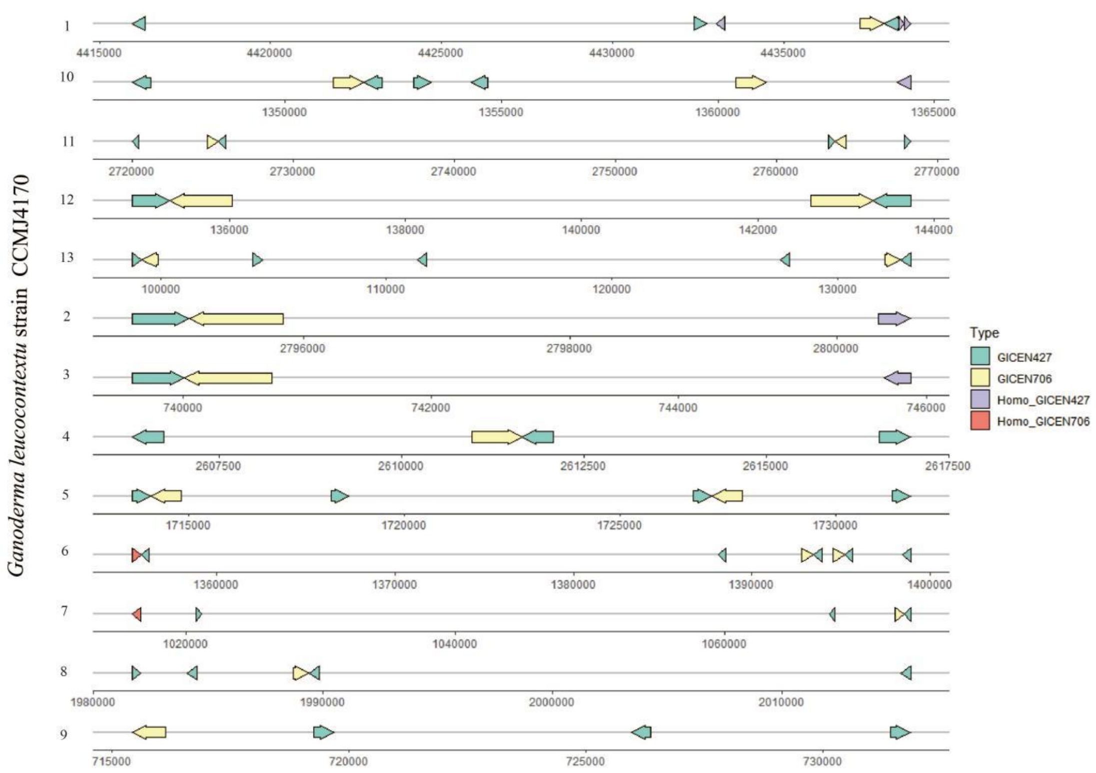
Ganoderma leucocontextu strain CCMJ4170
Type
GICEN427
GICEN706
Homo_GICEN427
Homo_GICEN706
1
10
11
12
13
2
3
4
5
6
7
8
9

Figure S7

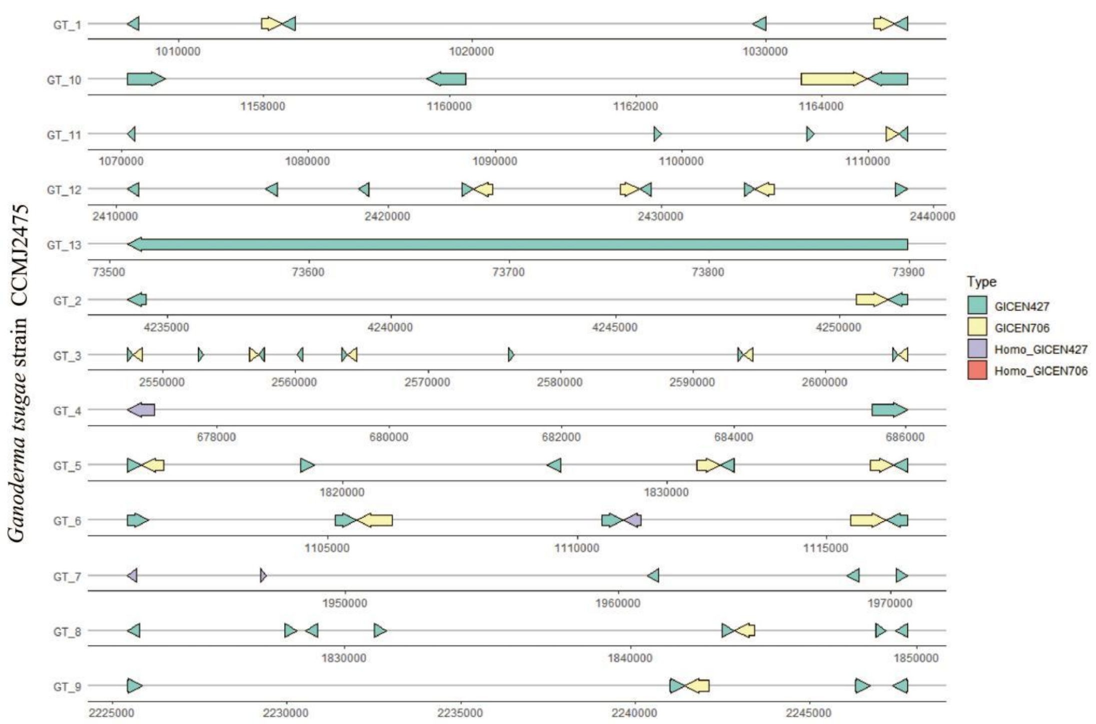

Ganoderma tsugae strain CCMJ2475
GT_1
1010000
1020000
1030000
GT_10
1158000
1160000
1162000
1164000
GT_11
1070000
1080000
1090000
1100000
1110000
GT_12
2410000
2420000
2430000
2440000
GT_13
73500
73600
73700
73800
73900
GT_2
4235000
4240000
4245000
4250000
GT_3
2550000
2560000
2570000
2580000
2590000
2600000
GT_4
678000
680000
682000
684000
686000
GT_5
1820000
1830000
GT_6
1105000
1110000
1115000
GT_7
1950000
1960000
1970000
GT_8
1830000
1840000
1850000
GT_9
2225000
2230000
2235000
2240000
2245000
Type
GICEN427
GICEN706
Homo_GICEN427
Homo_GICEN706

Figure S8

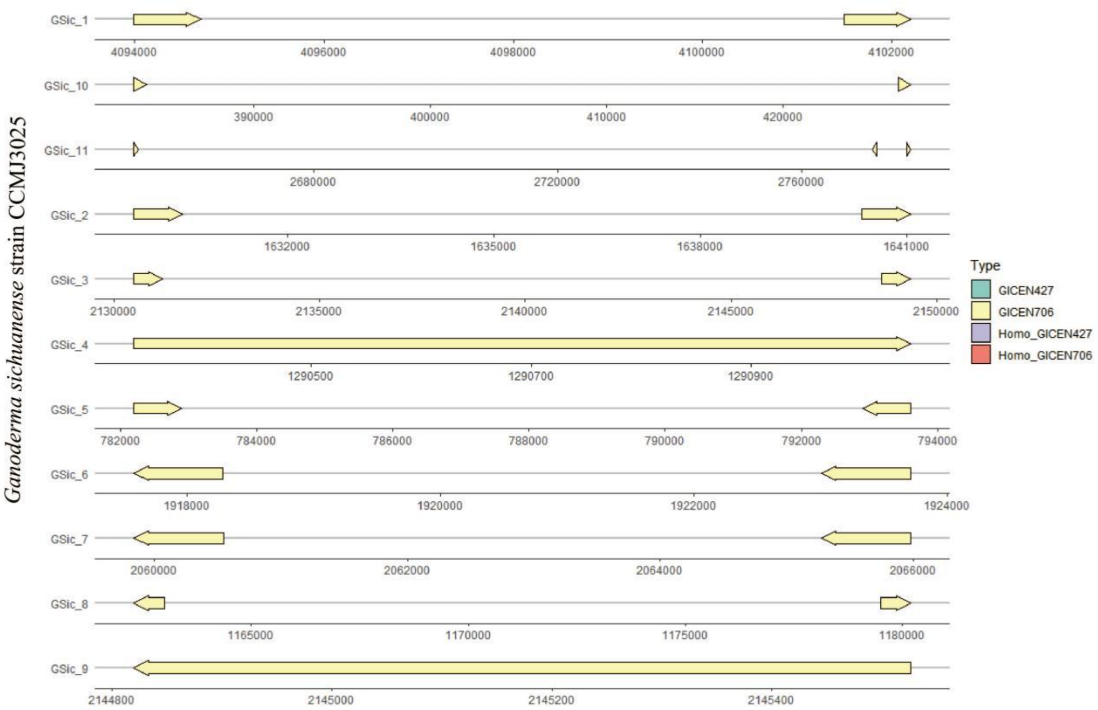
Ganoderma sichuanense strain CCMJ3025
GSic_1
4094000
4096000
4098000
4100000
4102000
GSic_10
390000
400000
410000
420000
GSic_11
2680000
2720000
2760000
GSic_2
1632000
1635000
1638000
1641000
GSic_3
2130000
2135000
2140000
2145000
2150000
GSic_4
1290500
1290700
1290900
GSic_5
782000
784000
786000
788000
790000
792000
794000
GSic_6
1918000
1920000
1922000
1924000
GSic_7
2060000
2062000
2064000
2066000
GSic_8
1165000
1170000
1175000
1180000
GSic_9
2144800
2145000
2145200
2145400
Type
GICEN427
GICEN706
Homo_GICEN427
Homo_GICEN706

Figure S9

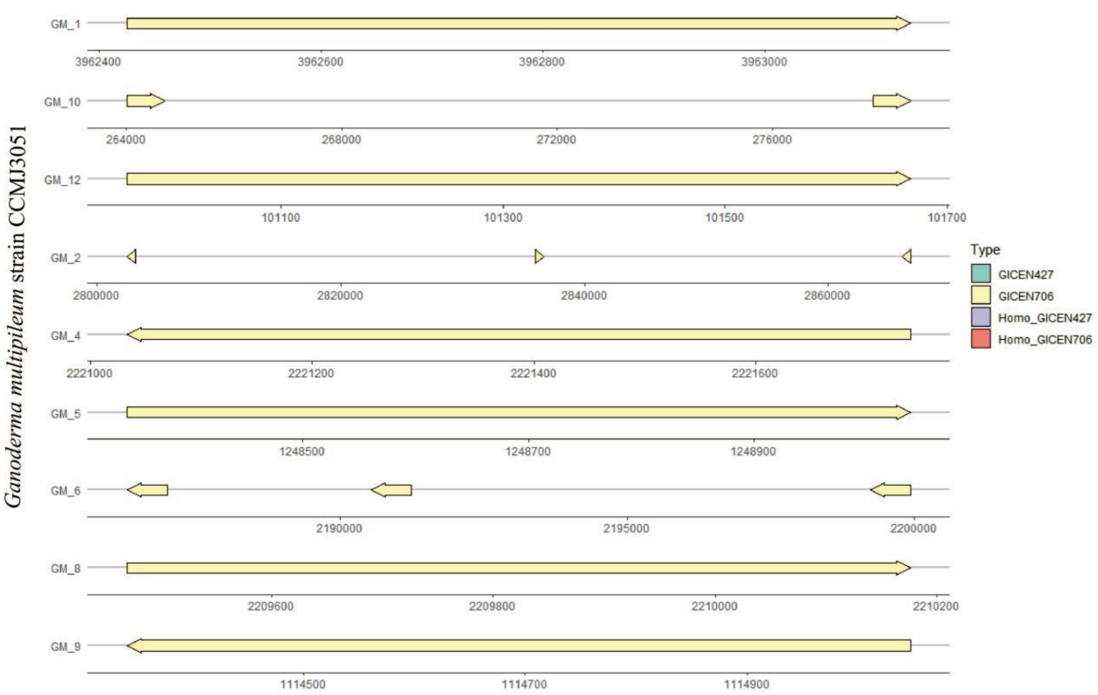
Ganoderma multipileum strain CCMJ3051
GM_1
3962400
3962600
3962800
3963000
GM_10
264000
268000
272000
276000
GM_12
101100
101300
101500
101700
GM_2
2800000
2820000
2840000
2860000
GM_4
2221000
2221200
2221400
2221600
GM_5
1248500
1248700
1248900
GM_6
2190000
2195000
2200000
GM_8
2209600
2209800
2210000
2210200
GM_9
1114500
1114700
1114900
Type
GICEN427
GICEN706
Homo_GICEN427
Homo_GICEN706

Figure S10

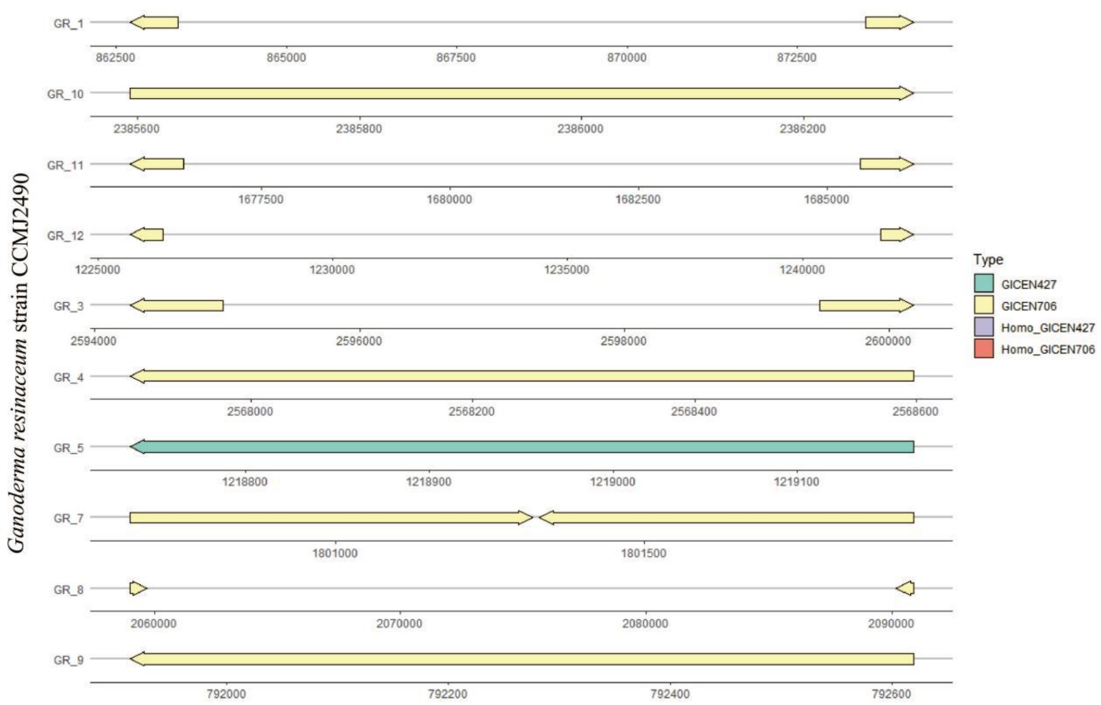
Ganoderma resinaceum strain CCMJ2490
GR_1
862500
865000
867500
870000
872500
GR_10
2385600
2385800
2386000
2386200
GR_11
1677500
1680000
1682500
1685000
GR_12
1225000
1230000
1235000
1240000
GR_3
2594000
2596000
2598000
2600000
GR_4
2568000
2568200
2568400
2568600
GR_5
1218800
1218900
1219000
1219100
GR_7
1801000
1801500
GR_8
2060000
2070000
2080000
2090000
GR_9
792000
792200
792400
792600
Type
GlCEN427
GlCEN706
Homo_GlCEN427
Homo_GlCEN706

Figure S11

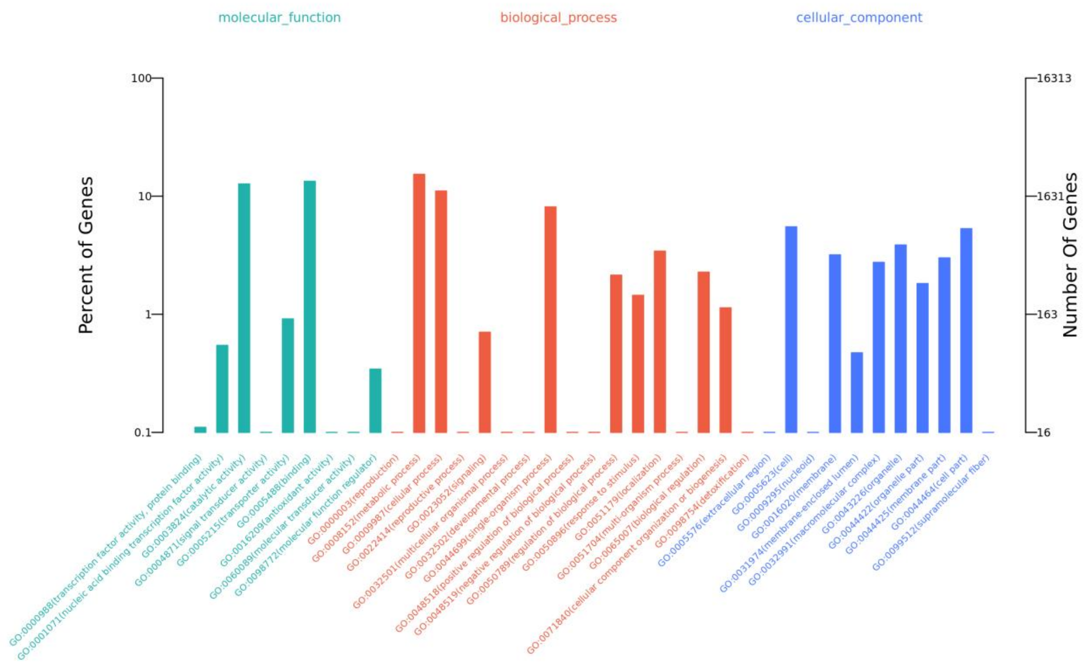
molecular_function
biological_process
cellular_component
Percent of Genes
Number Of Genes
100
10
1
0.1
16313
1631
163
16
GO:0000988(transcription factor activity, protein binding)
GO:0001071(nucleic acid binding transcription factor activity)
GO:0003824(catalytic activity)
GO:0004871(signal transducer activity)
GO:0005215(transporter activity)
GO:0005488(binding)
GO:0016209(antioxidant activity)
GO:0060089(molecular transducer activity)
GO:0098772(molecular function regulator)
GO:0000003(reproduction)
GO:0008152(metabolic process)
GO:0009987(cellular process)
GO:0022414(reproductive process)
GO:0023052(signaling)
GO:0032501(multicellular organismal process)
GO:0032502(developmental process)
GO:0044699(single-organism process)
GO:0048518(positive regulation of biological process)
GO:0048519(negative regulation of biological process)
GO:0050789(regulation of biological process)
GO:0050896(response to stimulus)
GO:0051179(localization)
GO:0051704(multi-organism process)
GO:0065007(biological regulation)
GO:0071840(cellular component organization or biogenesis)
GO:0098754(detoxification)
GO:0005576(extracellular region)
GO:0005623(cell)
GO:0009295(nucleoid)
GO:0016020(membrane)
GO:0031974(membrane-enclosed lumen)
GO:0032991(macromolecular complex)
GO:0043226(organelle)
GO:0044422(organelle part)
GO:0044425(membrane part)
GO:0044464(cell part)
GO:0099512(supramolecular fiber)

Figure S12

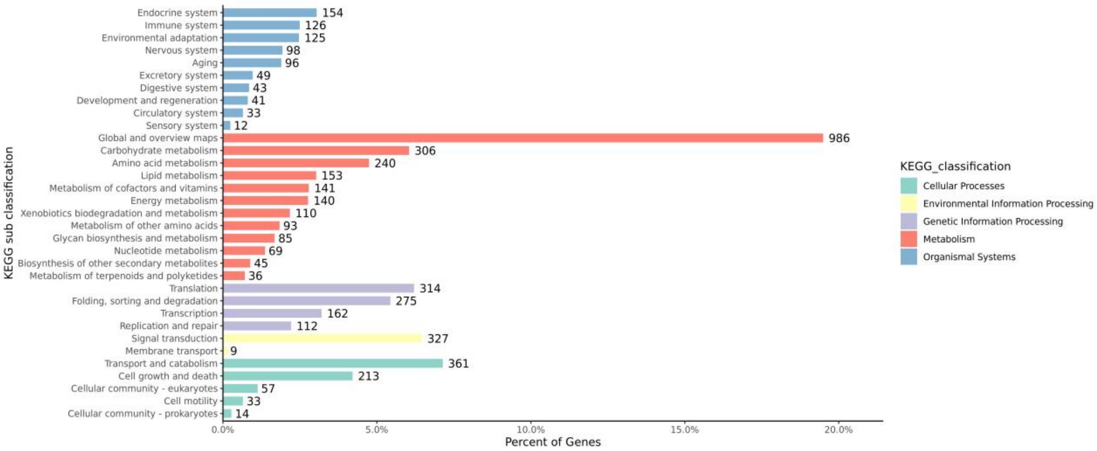
KEGG sub classification
Endocrine system
154
Immune system
126
Environmental adaptation
125
Nervous system
98
Aging
96
Excretory system
49
Digestive system
43
Development and regeneration
41
Circulatory system
33
Sensory system
12
Global and overview maps
986
Carbohydrate metabolism
306
Amino acid metabolism
240
Lipid metabolism
153
Metabolism of cofactors and vitamins
141
Energy metabolism
140
Xenobiotics biodegradation and metabolism
110
Metabolism of other amino acids
93
Glycan biosynthesis and metabolism
85
Nucleotide metabolism
69
Biosynthesis of other secondary metabolites
45
Metabolism of terpenoids and polyketides
36
Translation
314
Folding, sorting and degradation
275
Transcription
162
Replication and repair
112
Signal transduction
327
Membrane transport
9
Transport and catabolism
361
Cell growth and death
213
Cellular community - eukaryotes
57
Cell motility
33
Cellular community - prokaryotes
14
0.0%
5.0%
10.0%
15.0%
20.0%
Percent of Genes
KEGG_classification
Cellular Processes
Environmental Information Processing
Genetic Information Processing
Metabolism
Organismal Systems

Figure S13

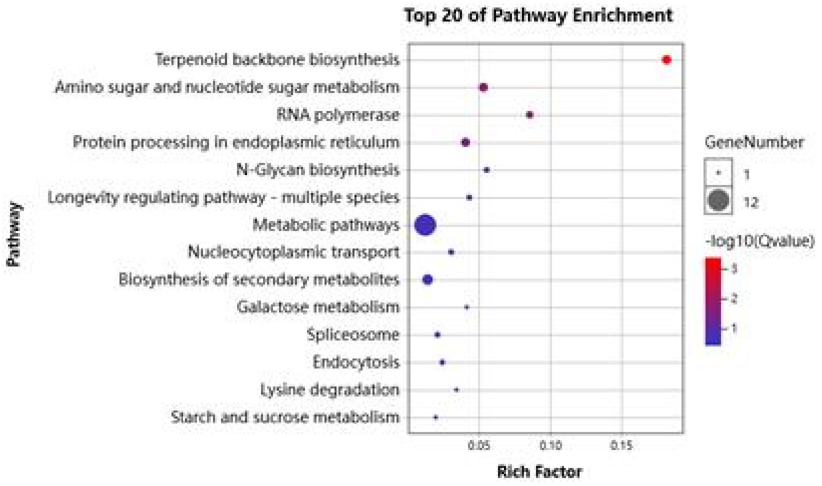
Top 20 of Pathway Enrichment
Terpenoid backbone biosynthesis
Amino sugar and nucleotide sugar metabolism
RNA polymerase
Protein processing in endoplasmic reticulum
N-Glycan biosynthesis
Longevity regulating pathway - multiple species
Metabolic pathways
Nucleocytoplasmic transport
Biosynthesis of secondary metabolites
Galactose metabolism
Spliceosome
Endocytosis
Lysine degradation
Starch and sucrose metabolism
Pathway
0.05
0.10
0.15
Rich Factor
GeneNumber
1
12
-log10(Qvalue)
3
2
1

Figure S14

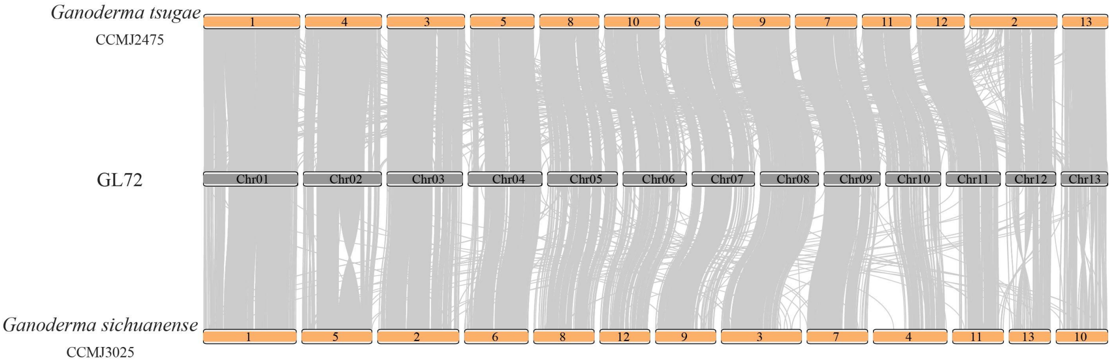

Ganoderma tsugae
CCMJ2475
1 4 3 5 8 10 6 9 7 11 12 2 13
GL72
Chr01 Chr02 Chr03 Chr04 Chr05 Chr06 Chr07 Chr08 Chr09 Chr10 Chr11 Chr12 Chr13
Ganoderma sichuanense
CCMJ3025
1 5 2 6 8 12 9 3 7 4 11 13 10

Figure S15

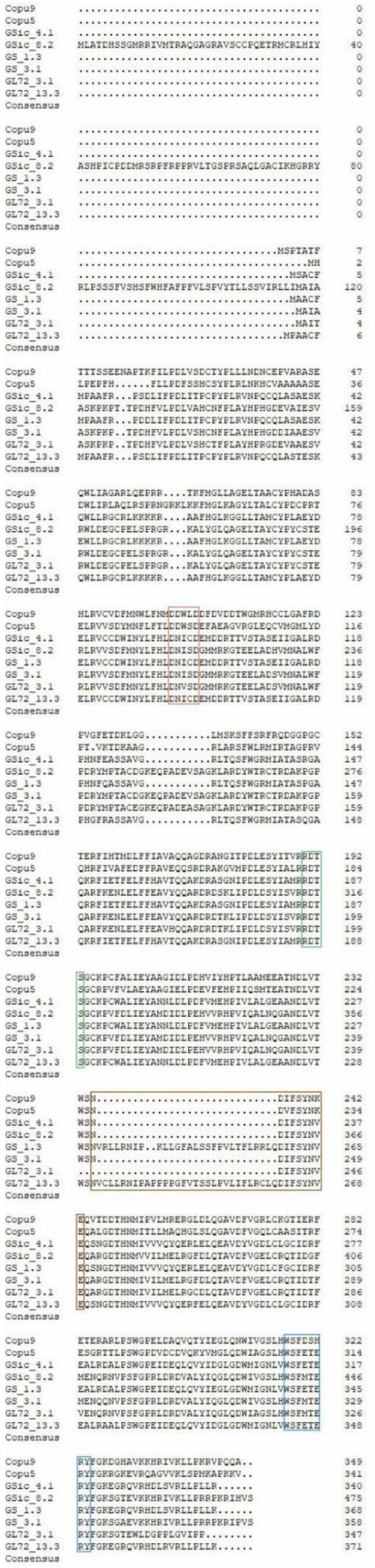

Table S1

Species	Strain	Numble	Terpene	Clavaric acid	Source
Ganoderma gibbosum	CCMJ3046	45	26	no	[9]
Ganoderma tsugae	CCMJ2475	34	16	yes	[9]
Ganoderma leucocontextum	GL72	39	18	yes	This study
Ganoderma resinaceum	CCMJ2490	41	22	yes	[9]
Ganoderma sinense	CCMJ2497	37	17	yes	[9]
Ganoderma applanatum	CCMJ3043	40	23	yes	[9]
Ganoderma multipileum	CCMJ3051	42	22	yes	[9]
Ganoderma australe	CCMJ3053	43	22	yes	[9]
Ganoderma sichuanense	CCMJ3025	37	19	yes	[9]
Ganoderma weberianum	CCMJ3054	47	26	yes	[9]
Ganoderma leucocontextum	CCMJ2485	37	20	yes	[9]
Amauroderma rugosum	ACCC 51706	36	16	yes	NCBI: GCA_017499575.1
Ganoderma lucidum	Ling-Jian NO.2	33	17	yes	NCBI: GCA_019426095.1
Ganoderma tsugae	s90	26	9	no	[9]
Ganoderma sinense	ZZ0214-1	40	20	yes	[53]
Ganoderma multipileum	BCRC 37180	45	23	yes	NCBI: GCA_000338015.1
Ganoderma boninense	G3	57	24	no	NCBI: GCA_002900995.2

Table S2

Chr	Size(bp)
Chr01	5,257,413
Chr02	4,353,368
Chr03	4,221,714
Chr04	4,115,428
Chr05	3,900,741
Chr06	3,539,945
Chr07	3,483,796
Chr08	3,239,031
Chr09	3,131,638
Chr10	3,066,788
Chr11	3,007,772
Chr12	2,781,080
Chr13	2,587,874
Total	46,686,588

Table S3

Type	Number	Percent (%)
Complete BUSCOs (C)	1,758	99.7
Complete and single-copy BUSCOs (S)	1,742	98.8
Complete and duplicated BUSCOs (D)	16	0.9
Fragmented BUSCOs (F)	2	0.1
Missing BUSCOs (M)	4	0.2
Total BUSCO groups searched	1,764	100

Table S4

**Chr numble**	**length**	**N counts**
1	5,518,212	200
2	4,901,690	200
3	4,240,300	300
4	4,087,960	100
5	3,644,271	400
6	3,391,645	200
7	3,310,795	200
8	3,189,542	200
9	3,106,008	200
10	2,872,700	0
11	2,856,557	300
12	2,727,109	400
13	2,678,846	100
Total	46,525,635	2800

Table S5

	GlCEN427	homo_GlCEN427	GlCEN706	homo_GlCEN706
Chr01	7	2	4	0
Chr02	2	3	1	0
Chr03	1	1	0	1
Chr04	7	0	6	0
Chr05	4	2	2	0
Chr06	1	2	1	0
Chr07	5	0	1	0
Chr08	3	0	1	0
Chr09	4	0	0	0
Chr10	3	3	0	4
Chr11	2	0	1	0
Chr12	8	1	3	0
Chr13	6	0	2	0
Total	53	14	22	5

Table S6

Chr	source	start	end	Type	RT	RH	IN	Gag-protein	Transposase	Transcriptional reads
Chr04	LTR_finder	2084675	2090288	Copia	RT_Tto1	RNaseH_Vitico1-2	INT_SmCIN1_Repbaseaccession_Polinton-1_SM	GAG_STcLV2PP1664	NA	0
Chr01	LTR_finder	1219554	1226567	Copia	RT_marY1	RNaseH_marY1	INT_Amn-ichi	GAG_Amn-ni	TR_ApGINGER1c	0
Chr01	LTR_havest	1257062	1262139	Copia	RT_Tto1	RNaseH_CopiaSL_23	INT_Tricopia	GAG_V12	TR_NvGINGER2-2_Repbaseaccession_Ginger2-2_NV	2
Chr01	LTR_havest	1308809	1315573	Copia	RT_Tto1	RNaseH_CopiaSL_23	INT_Tricopia	NA	TR_NvGINGER2-2_Repbaseaccession_Ginger2-2_NV	15
Chr02	LTR_havest	678987	682004	Copia	RT_marY1	RNaseH_marY1	INT_Amn-ichi	NA	TR_ApGINGER1c	0
Chr04	LTR_havers	2074144	2079917	Copia	RT_Tto1	RNaseH_Vitico1-2	INT_Tricopia	GAG_V12	NA	6

	t									
Chr05	LTR_haverst	2485786	2499251	Copia	RT_marY1	RNaseH_CopiaSL_23	INT_Tricopia	NA	NA	38
Chr06	LTR_haverst	2057204	2065722	Copia	RT_Xanthias	RNaseH_Vitico1-2	INT_Tricopia	GAG_HIV-1	TR_NvGINGER2-2_Repbaseaccession_Ginger2-2_NV	0
Chr07	LTR_haverst	2251481	2258988	Copia	RT_Tto1	RNaseH_Vitico1-2	INT_Tricopia	GAG_V12	TR_NvGINGER2-2_Repbaseaccession_Ginger2-2_NV	0
Chr08	LTR_haverst	2428819	2440315	Copia	RT_Batata	RNaseH_Vitico1-2	INT_Oryco1-1	GAG_Xanthias	NA	0
Chr09	LTR_haverst	2031986	2035087	Copia	NA	NA	NA	NA	NA	0
Chr10	LTR_haverst	1320816	1325469	Copia	RT_Tto1	NA	INT_Tricopia	NA	TR_NvGINGER2-2_Repbaseaccession_Ginger2-2_NV	0

Chr10	LTR_haverst	1329289	1333141	Copia	NA	RNaseH_CopiaSL_23	INT_Tor1	NA	TR_NvGINGER2-2_Repbaseaccession_Ginger2-2_NV	0
Chr11	LTR_haverst	2915460	2920571	Copia	RT_To1	RNaseH_CopiaSL_23	INT_Tricopia	GAG_Osvaldo	TR_NvGINGER2-2_Repbaseaccession_Ginger2-2_NV	0
Chr12	LTR_haverst	2614314	2620097	Copia	RT_To1	RNaseH_CopiaSL_23	INT_Tricopia	GAG_V12	TR_NvGINGER2-2_Repbaseaccession_Ginger2-2_NV	9
Chr12	LTR_haverst	2678831	2684614	Copia	RT_To1	RNaseH_CopiaSL_23	INT_Tricopia	GAG_V12	TR_NvGINGER2-2_Repbaseaccession_Ginger2-2_NV	41
Chr13	LTR_haverst	98516	104295	Copia	RT_To1	RNaseH_CopiaSL_23	INT_Tricopia	GAG_V12	TR_NvGINGER2-2_Repbaseaccession_Ginger2-2_NV	87

Table S7

Gene set	Total number of genes	Average gene length(bp)	Average CDS length(bp)	Average exons number per gene	Average exon length(bp)	Average intron length(bp)
De novo	12,353	2,158.07	1,435.21	7.04	204	119.77
Homology	9,994	1,871.60	1,459.69	5.67	257.43	88.2
RNA-seq	8,456	1,910.20	1,489.91	5.82	256.02	87.21
Final set	12,493	2,125.25	1,433.83	6.6	217.23	123.46

Table S8

Term Name	Type	MainClass	Gene Hits In Selected Set	All Genes In Background	p-value	enrichFactor
Nicotinate and nicotinamide metabolism	Expanded	A09100 Metabolism	7	3651	1.36557E-14	109.2179487
Organismal Systems	Expanded	A09150 Organismal Systems	7	3651	6.68132E-13	66.03875969
Longevity regulating pathway - multiple species	Expanded	A09150 Organismal Systems	7	3651	6.68132E-13	66.03875969
Aging	Expanded	A09150 Organismal Systems	7	3651	6.68132E-13	66.03875969
DNA replication proteins	Expanded	A09180 Brite Hierarchies	7	3651	1.77407E-10	30.86594203
Metabolism of cofactors and vitamins	Expanded	A09100 Metabolism	7	3651	3.92726E-09	19.99765258
Chromosome and associated proteins	Expanded	A09180 Brite Hierarchies	7	3651	1.516E-06	8.527527528

Metabolism	Expanded	A09100 Metabolism	9	3651	7.17159E-06	3.717922607
Purine metabolism	Expanded	A09100 Metabolism	2	3651	0.005513403	17.26241135
Nucleotide metabolism	Expanded	A09100 Metabolism	2	3651	0.011632321	11.75845411
Biosynthesis of various plant secondary metabolites	Contracted	A09100 Metabolism	2	3651	0.001797277	30.68067227
Cyanoamino acid metabolism	Contracted	A09100 Metabolism	2	3651	0.002664057	25.26643599
Amino sugar and nucleotide sugar metabolism	Contracted	A09100 Metabolism	3	3651	0.004431714	8.706677266
Biosynthesis of other secondary metabolites	Contracted	A09100 Metabolism	2	3651	0.008223315	14.31764706
Carbohydrate metabolism	Contracted	A09100 Metabolism	5	3651	0.010516415	3.520732883
Starch and sucrose metabolism	Contracted	A09100 Metabolism	2	3651	0.022760378	8.422145329
Enzymes with EC numbers	Contracted	A09190 Not Included in Pathway or	3	3651	0.026732003	4.50555327

		Brite				
Unclassified: metabolism	Contracted	A09190 Not Included in Pathway or Brite	3	3651	0.032937575	4.156736243
Cytochrome P450	Contracted	A09180 Brite Hierarchies	1	3651	0.041178539	23.8627451

Table S9

Species	Strain	Numble	Terpene	Clavaric acid	Source
Ganoderma gibbosum	CCMJ3046	45	26	no	Jiang et al., 2022
Ganoderma tsugae	CCMJ2475	34	16	yes	Jiang et al., 2021
Ganoderma leucocontextum	GL72	39	18	yes	
Ganoderma resinaceum	CCMJ2490	41	22	yes	Jiang et al., 2022
Ganoderma sinense	CCMJ2497	37	17	yes	Jiang et al., 2022
Ganoderma applanatum	CCMJ3043	40	23	yes	Jiang et al., 2022
Ganoderma multipileum	CCMJ3051	42	22	yes	Jiang et al., 2022
Ganoderma australe	CCMJ3053	43	22	yes	Jiang et al., 2022
Ganoderma sichuanense	CCMJ3025	37	19	yes	Jiang et al., 2022
Ganoderma weberianum	CCMJ3054	47	26	yes	Jiang et al., 2022
Ganoderma leucocontextum	CCMJ2485	37	20	yes	Jiang et al., 2022
Amauroderma rugosum	ACCC 51706	36	16	yes	Lin et al.,2021
Ganoderma lucidum	Ling-Jian NO.2	33	17	yes	NCBI:GCA_019426095.1
Ganoderma tsugae	s90	26	9	no	Jiang et al., 2021
Ganoderma sinense	ZZ0214-1	40	20	yes	Zhu et al.,2015
Ganoderma multipileum	BCRC 37180	45	23	yes	NCBI:GCA_000338015.1
Ganoderma boninense	G3	57	24	no	NCBI:GCA_002900995.2

Table S10

Clust numble	Clust typle	Gene	Gene typle
Chr02.region005	terpene	LG02.1229	other gene
		LG02.1230	other gene
		LG02.1231	core biosynthetic genes
		LG02.1232	other gene
		LG02.1234	other gene

Table S11

Gene	Chromosome	Start	End	E-value	Rate
LG12.14	Chr12	71629	74031	1.30E-27	2.575582148
LG01.54	Chr01	236399	233967	0.0076	4.496489053
LG04.20	Chr04	195737	190760	1.40E-38	4.756176028
LG08.50	Chr08	158552	151394	6.10E-27	4.895044228
LG08.82	Chr08	263746	260316	2.40E-146	8.142743926
LG07.106	Chr07	367187	364660	3.90E-13	10.5395602
LG01.139	Chr01	647357	649641	3.80E-34	12.31322325
LG03.190	Chr03	572666	571115	2.50E-29	13.56477488
LG09.158	Chr09	524981	529871	5.80E-11	16.76378304
LG13.107	Chr13	541327	543315	1.10E-33	20.91782676
LG03.313	Chr03	942710	948717	6.60E-19	22.33002994
LG08.249	Chr08	860785	863221	3.30E-12	26.57538628
LG03.395	Chr03	1155819	1153370	9.10E-17	27.37795597
LG03.428	Chr03	1266514	1264899	9.00E-26	29.99999526
LG01.340	Chr01	1586772	1584643	2.20E-23	30.18161214
LG01.342	Chr01	1589311	1591493	2.70E-30	30.22990585
LG03.461	Chr03	1344330	1347177	2.10E-32	31.84322766
LG13.199	Chr13	846972	843901	3.30E-16	32.72848678
LG01.507	Chr01	2065900	2063949	2.30E-22	39.29499166
LG01.527	Chr01	2109930	2113800	5.50E-23	40.1324758
LG02.564	Chr02	1842557	1840147	2.60E-22	42.32486204
LG13.273	Chr13	1100314	1098656	4.40E-30	42.51806695
LG12.266	Chr12	1232736	1231210	1.30E-61	44.32580149
LG01.701	Chr01	2558809	2552004	8.20E-26	48.67049631
LG03.718	Chr03	2062791	2057848	2.30E-25	48.86145769
LG01.793	Chr01	2802334	2798533	2.40E-18	53.30252731
LG01.816	Chr01	2849239	2846309	1.50E-119	54.19469614
LG07.534	Chr07	1897708	1895051	2.90E-22	54.47090367
LG08.539	Chr08	1770691	1774453	8.00E-11	54.66730636
LG09.537	Chr09	1772850	1768809	2.50E-154	56.61094929
LG02.794	Chr02	2511794	2513437	4.00E-28	57.69771818
LG03.851	Chr03	2459180	2455125	9.10E-152	58.25074839
LG03.858	Chr03	2477620	2475371	1.80E-32	58.68753781
LG02.820	Chr02	2577268	2573624	4.90E-38	59.20170314
LG02.826	Chr02	2597124	2591163	1.90E-21	59.65780977
LG03.882	Chr03	2532323	2529419	1.20E-06	59.98329115
LG02.856	Chr02	2663603	2669595	1.30E-11	61.1848803
LG04.648	Chr04	2822270	2819946	4.80E-12	68.57780041
LG07.661	Chr07	2399006	2394465	3.40E-35	68.85992193
LG04.674	Chr04	2902143	2905325	2.20E-14	70.51861921
LG01.1156	Chr01	3771368	3776246	4.40E-11	71.73429213

LG05.686	Chr05	2804589	2806725	5.80E-28	71.89887767
LG03.1078	Chr03	3284942	3279870	5.30E-13	77.81062384
LG13.574	Chr13	2054157	2052052	9.00E-27	79.37623702
LG04.836	Chr04	3397889	3408414	2.00E-49	82.5646567
LG05.779	Chr05	3342965	3339167	8.80E-19	85.70076814
LG02.1152	Chr02	3771552	3769080	2.50E-20	86.63526722
LG03.1199	Chr03	3697429	3699820	5.90E-31	87.58122886
LG03.1202	Chr03	3710275	3707884	2.70E-31	87.88551285
LG06.766	Chr06	3141922	3144786	1.20E-66	89.81811238
LG06.798	Chr06	3426035	3421857	6.00E-19	97.94004964
